# Control of Solid-Supported Intra- vs Interstrand Stille Coupling Reactions for Synthesis of DNA–Oligophenylene Conjugates

**DOI:** 10.1021/acs.bioconjchem.4c00310

**Published:** 2024-07-24

**Authors:** Chu-Fan Yang, Thanuka Udumulla, Ruojie Sha, James W. Canary

**Affiliations:** Department of Chemistry, New York University, New York, New York 10003, United States

## Abstract

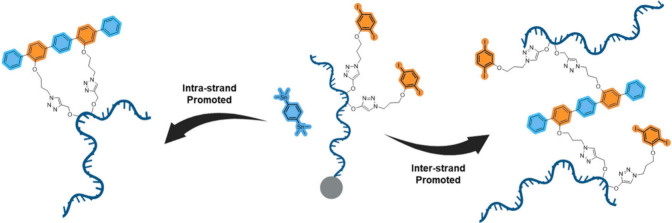

Programmed DNA structures and assemblies are readily accessible, but site-specific functionalization is critical to realize applications in various fields such as nanoelectronics, nanomaterials and biomedicine. Besides pre- and post-DNA synthesis conjugation strategies, on-solid support reactions offer advantages in certain circumstances. We describe on-solid support internucleotide coupling reactions, often considered undesirable, and a workaround strategy to overcome them. Palladium coupling reactions enabled on-solid support intra- and interstrand coupling between single-stranded DNAs (ss-DNAs). Dilution with a capping agent suppressed interstrand coupling, maximizing intrastrand coupling. Alternatively, interstrand coupling actually proved advantageous to provide dimeric organic/DNA conjugates that could be conveniently separated from higher oligomers, and was more favorable with longer terphenyl coupling partners.

With a few simple tools, structural DNA nanotechnology allows the design and assembly of an unparalleled variety of programmed molecular structures and assemblies.^1−3^ Covalent conjugation of organic molecules to DNA imparts new functions to DNA assemblies: DNA modified with functional groups create intricate three-dimensional DNA nanostructures.^3,4^ Synthetic methodology toward functionalized DNA nanostructures should amplify the impact of this field.

Covalent DNA conjugates are formed using synthetic nucleoside analogue reagents in automated DNA synthesis (a “pre-strategy”), or by chemical reaction on natural or postsynthetic DNA molecules (“post-strategy”).^5−10^ The “pre-strategy” usually suffers from low yield of both multistep synthesis and coupling into DNA sequences, while the “post- strategy” is often impeded by not only difficult purification, but also low coupling yield and incompatibility to reaction conditions.^11,12^ Alternatively, “on solid support” palladium-catalyzed coupling reactions functionalize nucleotides and oligonucleotides *during* nucleoside oligomerization, while the incipient ssDNA molecule is still attached to the solid support,^11−15^ providing easier synthesis and purification, tolerance to harsh reaction conditions, as well as avoiding difficulties in cross-coupling of dsDNA.^16−23^ (We note that some authors classify the on solid support strategy together with other postsynthetic methods.^24^) Polymer templation by DNA sequences attracts our interest for control of dispersity and topology.^25,26^ However, interstrand coupling may occur on solid support to produce mixtures with bifunctional reagents. Here, we explore inter- and intra- strand coupling, and find that we can control the outcome of the reaction to achieve the desired aim (Figure 1).

**Figure 1:**
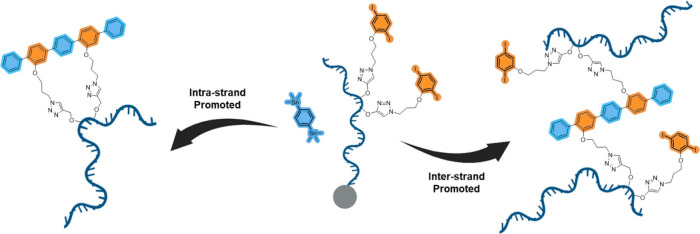
On-solid support inter- and intrastrand coupling reactions.

Previously, we synthesized several monodisperse organic oligomers on DNA. We combined pre- and poststrategies to prepare nylon nucleic acids.^25,27,28^ The same technology created a variety of templated linkages within and between DNA molecules.^29−32^ When 2′-propargyl phosphoramidite reagents became commercially available, we constructed DNA-oligoaniline^26,33,34^ and DNA-oligophenylenevinylene^26,35^ conjugates on the solid support with a synthetic step in the middle of the automated DNA synthesis. We observed on-solid support interstrand coupling of bifunctional molecules^26^ by polyacrylamide gel electrophoresis. We previously used a desymmetrization strategy to avoid interstrand coupling.^34^ A recent publication did not report interstrand coupling in a similar scenario.^36^

In this paper, we fabricate DNA-polyparaphenylene ladder oligomers (DNA-PPPs). Known for remarkable stiffness and strength, PPPs could provide strong reinforcement to DNA nanostructures similar to DNA-silica hybrid materials.^37^ An important material in organic electronics, PPP’s bipolar doping capability, stability, and other properties lead to its application in batteries and other applications.^38−40^ PPP is difficult to synthesize by direct polymerization routes due to insolubility of even short oligomers.^41^

We envisaged a sequence of automatic DNA synthesis, conjugation with exogenous reagents, and palladium-catalyzed coupling in solid phase to access DNA-PPP conjugates. We prepared solid supported propargyl-modified single-strand DNAs (ss-DNAs) with commercially available solid supports and nucleoside phosphoramidites. A multistep synthetic sequence gave building blocks with azide groups (Supporting Information (SI), Figure S1), which were subsequently subjected to copper-catalyzed azide–alkyne cycloaddition (CuAAC reaction, known as “click reaction”)^42−44^ with previously prepared propargyl-modified ss-DNAs. MALDI-TOF characterization confirmed the complete conversion by click reaction and formation of DNA iodides on two different solid supports (SI, Table S1).

We chose to perform aryl–aryl coupling reactions^45^ on-solid support to leverage the benefits of solid-phase chemistry.^46,47^ Different named reactions (Suzuki, Stille, etc.) describe reaction conditions used in Pd coupling reactions. We initially worked with organoboron reagents (Suzuki) but found the required base reagents to be incompatible with basic-cleavable CPG solid support with the standard long chain alkylamino (lcaa) linker. Suzuki conditions worked better with basic-uncleavable oligo affinity support polystyrene (OAS PS) solid support, but ultimately the organotin reagents (Stille) allowed better optimization.

We examined a DNA sequence containing a phenyl monoiodide moiety reacting with phenyltrialkyltin (SI, Table S2). We probed solid phase coupling reaction conditions with phenyltrialkyltin, catalyzed by Pd_2_dba_3_ and AsPh_3_ in DMF. MALDI-TOF analysis indicated a failed coupling reaction with commonly used phenyltributyltin coupling partner. By contrast, phenyltrimethyltin gave complete conversion, consistent with the superiority of trimethyltin over its tributyl analogue in transmetalation, typically the rate-determining step in the Stille coupling reaction.^48−50^ Besides trimethyltin species, the soluble copper(I) complex Cu(CH_3_CN)_4_PF_6_ also accelerated the on-solid support Stille reactions.^50−52^ With successful coupling between monoiodide building blocks and phenyltrimethyltin, we turned our attention to the coupling reaction involving 1,4-bis(trimethylstannyl)benzene (C_1_), which is expected to provide both intra- and interstrand coupling products (Figure 1). The anticipated pentaphenyl species (intrastrand product, “monomer”) was observed clearly as a significant product (SI, Figure S15) in MALDI-TOF analysis, accompanied by a minor bis(terphenyl) byproduct.

PAGE (polyacrylamide gel electrophoresis) was used to further analyze the reaction mixture to investigate high mass species that are difficult to observe for MALDI-TOF. PAGE indicated that for reactions involving doubly and triply modified DNA strands producing bands with lower mobilities, corresponding to high-mass species (interstrand products).

These bands were consistent with the generation of oligomers via on-solid support, intermolecular coupling (Figure 2a). The coupling reaction between monoiodide moieties and bis(trimethylstannyl) reagent theoretically yields monomer and dimer products, which aligned with the results observed in the PAGE analysis (Figure 2b, lane 2). Introducing diiodide building blocks allowed for the formation of oligomers through continuous intermolecular coupling, as demonstrated in Lane 3 (Figure 2b).

**Figure 2:**
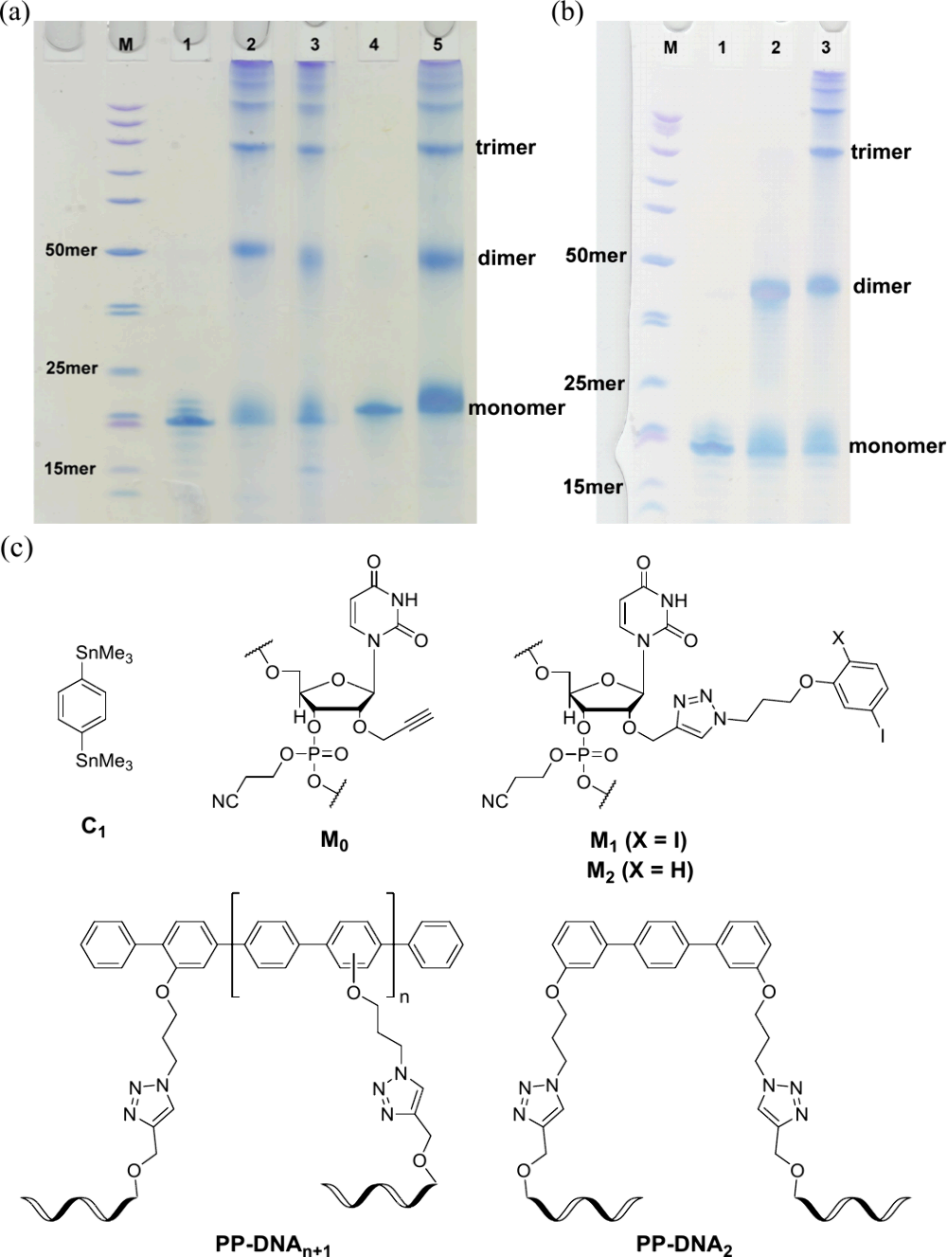
The 20% denaturing polyacrylamide gel electrophoresis (PAGE). (a) Lane M: standard DNA size markers. Lane 1: S_1_: 5′-TTT M_1_M_1_T TTT TTT TTT TTT-3′. Lane 2: coupling product (PP-DNA_*n*+1_) of S_1_. Lane 3: coupling product (PP-DNA_*n*+1_) of S_2_: 5′-TTT M_1_TT M_1_TT TTT TTT TTT-3′. Lane 4: S_3_: 5′-TM_1_T M_1_TM_1_ TTT TTT TTT TTT-3′. Lane 5: coupling product (PP-DNA_n+1_) of S_3_. (b) Lane M: standard DNA size markers. Lane 1: S_4_: 5′-TTT M_0_TT TTT TTT TTT TTT-3′. Lane 2: coupling product (PP-DNA_2_ of S_5_: 5′-TTT M_2_TT TTT TTT TTT TTT-3′. Lane 3: coupling product (PP-DNA_2_ of S_6_: 5′-TTT M_1_TT TTT TTT TTT TTT-3′. (c) Coupling partner C_1_. Modified uridines: M_*0*_, M_1_ and M_2_. Structures of interstrand products PP-DNA_*n*+1_ and PP-DNA_2_.

Our attempts to identify those oligomers by mass spectrometry failed, consistent with our inability to observe high mass DNA conjugates by MALDI-TOF. As an alternative, we confirmed the structures of the oligomers by characterizing the coupling reactions of short T12 strands using 1,4-bis(trimethylstannyl)benzene as the coupling partner. Major peaks corresponding to monomers were found at 3960 and 4035 Da, respectively (Figure 3). Weaker but distinct signals representing dimers were detected at 7845 Da for the monoiodide strand and 7995 Da for the diiodide species. The pentaphenyl product may be formed by a variety of sequences of reactions as illustrated in SI, Figure S16. Two proximal aryl iodides may be bridged by a phenyl bis(trimethyltin) reagent. The remaining aryl iodides may react with excess phenyl bis(trimethyltin) reagent to produce pentamer terminated by trimethyltin substituents. Protodestannylation under the reaction conditions provides the observed product. Three regioisomers of products are likely to be formed, although we did not attempt to separate them.

**Figure 3:**
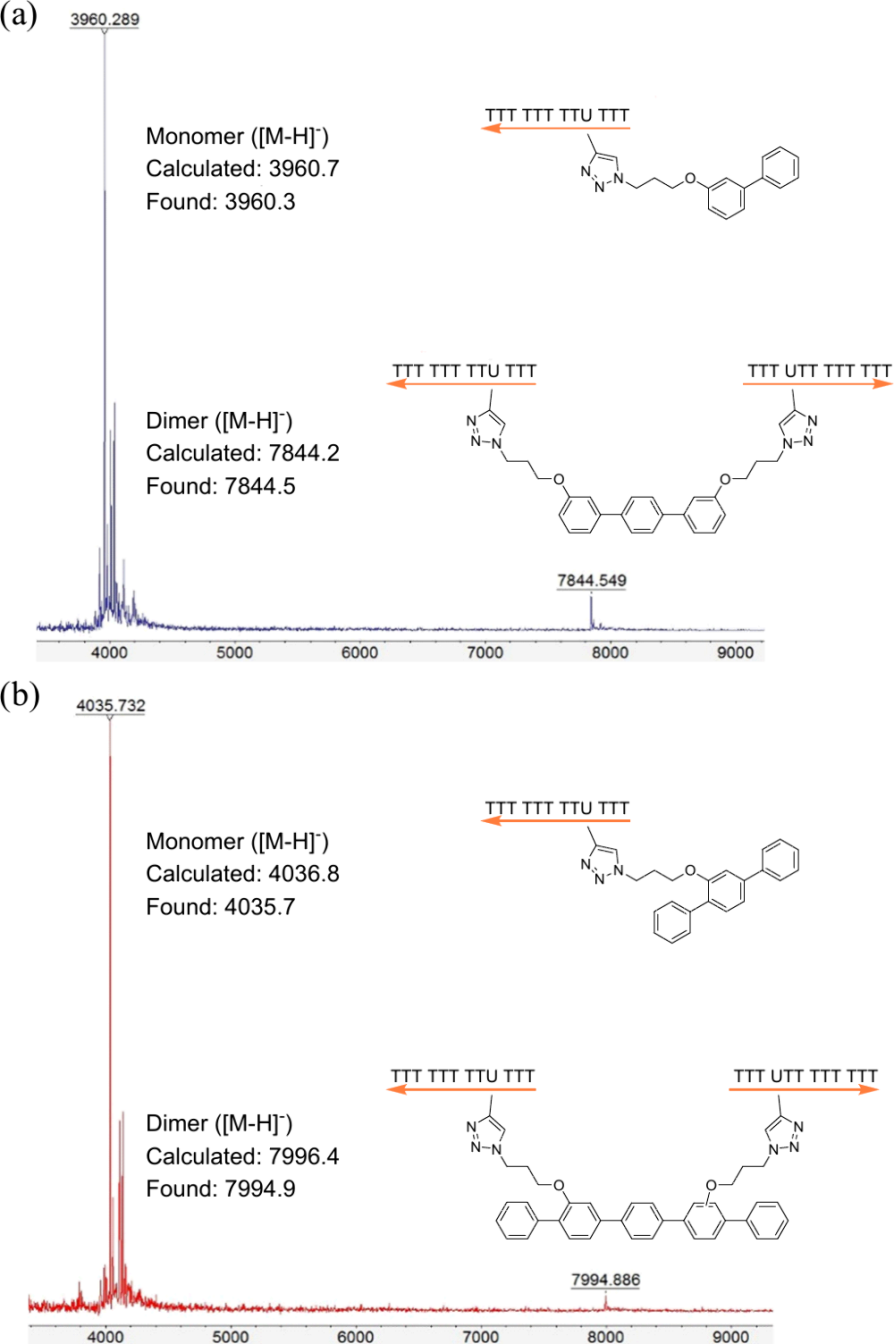
(a) DNA strand: 5′-TTT M_2_TT TTT TTT-3′. (b) DNA strand: 5′-TTT M_1_TT TTT TTT-3′. Coupling partner C_1_.

Coupling reactions on singly or doubly modified heterobase strands, consisting of all four ATCG nucleotides was also investigated and observed to undergo interstrand coupling successfully (SI, Figure S17). As shown in Figure 4a, monomers of both heterobase (HB) strands HB-S_1_ and HB-S_2_ annealed with complementary strands to form duplexes, indicated by their slower mobility. The high conversion was confirmed by disappearance of both the monomer bands and the complementary bands. The same scenario was observed for dimers (Figure 4b), indicating that heterobase strands remain viable for hybridization after coupling reactions.

**Figure 4:**
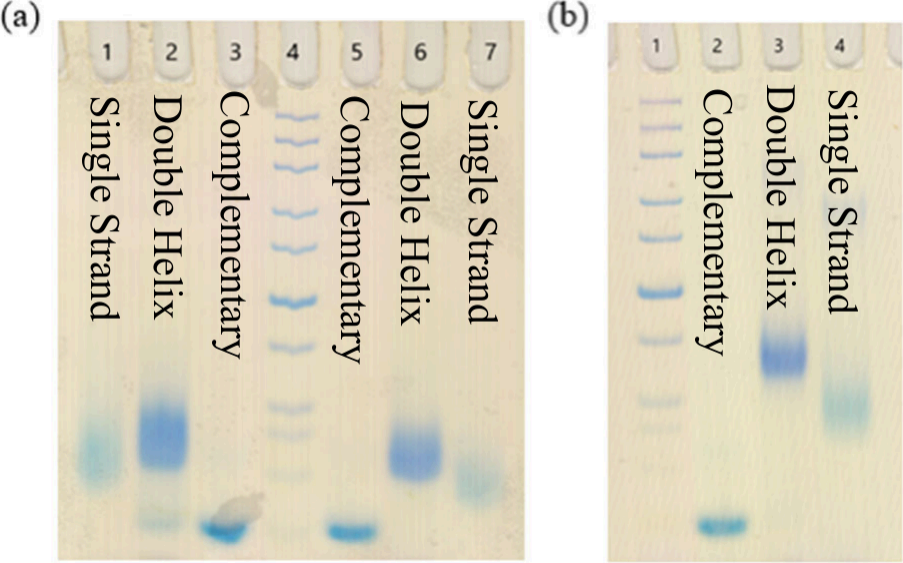
The 10% nondenaturing PAGE. (a) Lane 1: HB-S_2_ monomer. Lane 2: duplex of HB-S_2_ monomer with its complement. Lane 3: complementary strand. Lane 4: standard marker. Lane 5: complementary strand. Lane 6: duplex of HB-S_1_ monomer with its complement. Lane 7: HB-S_1_ monomer. (b) Lane 1: standard marker. Lane 2: complementary strand. Lane 3: duplex of HB-S_2_ dimer with its complement. Lane 4: HB-S_2_ dimer. ss-DNA strands: HB-S_1_: 5′-M_1_TG CAG TCT TTT TT-3′; HB-S_2_: 5′- M_1_TM_1_ GCA GTC TTT TTT-3′, Complementary strand: 5′-AAA AAA GAC TGC AAA-3′.

To obtain intrastrand coupling, we devised a strategy to “dilute” the DNA strands on solid support by capping them with phosphoramidite without 5′-hydroxy groups and reduce the statistical proximity of the aryl iodides. Figure 5a shows the design by diluting solid supported oligodeoxynucleotides (ODNs) with a biotin additive end-cap. During automated DNA synthesis, a mixture of 90% 5′-biotin and 10% thymidine was used instead of 100% thymidine. This approach aimed to generate a majority of terminated single-stranded DNA ODNs, which lack any reactive sites for the Stille reaction.

**Figure 5:**
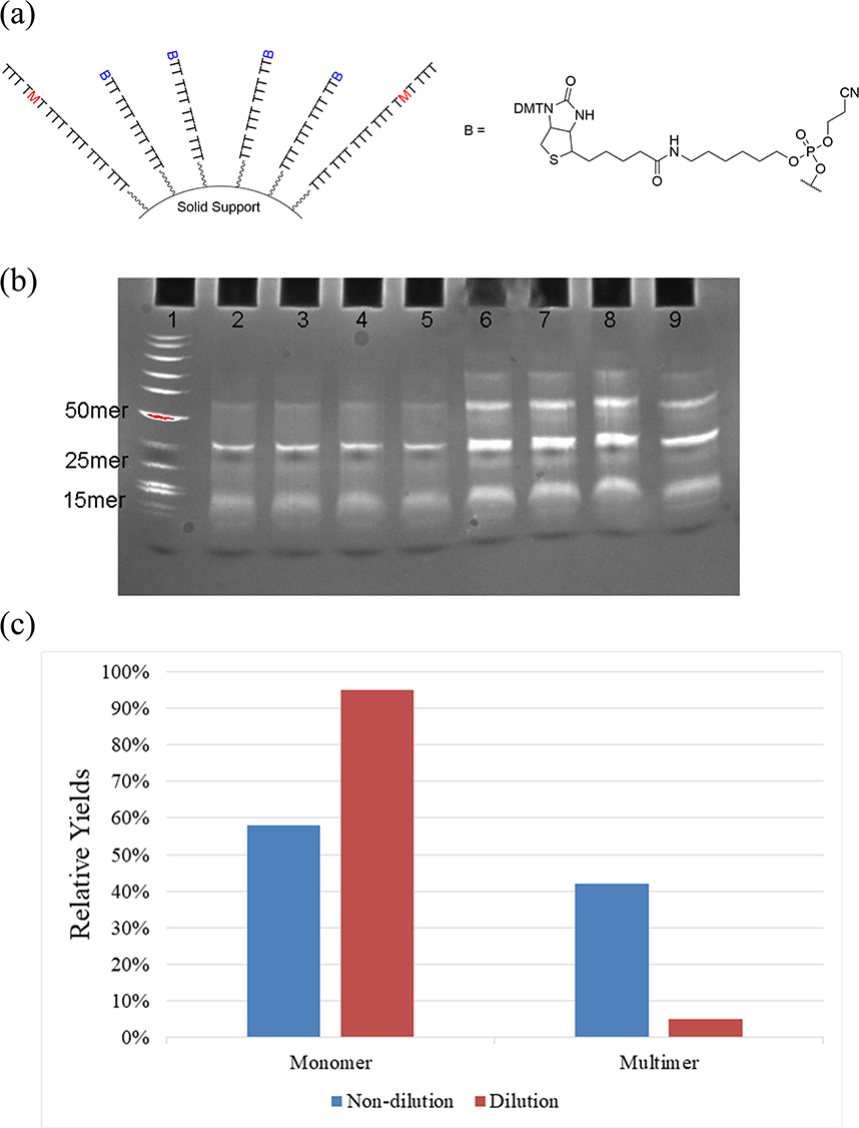
(a) Dilution of ss-DNA on solid support. (b) 20% denaturing PAGE: Lane 1: standard markers. Lane 2 to 5: coupling products of S_7_ and C_1_. Lane 6 to 9: coupling products of S_8_ and C_1_. (c) After gel purification, OD_260_ measurement gave relative yields of monomer vs the sum of multimers: with 100% T, the ratio of monomer:dimer:trimer = 58%:30%:12%; with 90% 5′-biotin and 10% T: the ratio of multimers <5%. ss-DNA strands: S_7_: 5′-TTT M_1_TM_1_ TTT XTT TTT TTT-3′; S_8_: 5′-TTT M_1_TM_1_ TTT TTT TTT TTT-3′; X: amidites mixtures (90% 5′-biotin and 10% T).

We hypothesized that the remaining normal ODNs, not terminated by biotin, would have greater distances between them compared to a typical solid support sample. We conducted polyacrylamide gel electrophoresis (PAGE) analysis of the coupling reactions using these biotin-diluted ODNs. Various diluted ODNs were examined, and the results (Figure 5b) showed minimal material in the high mass range and only a few dimers compared to the reactions using normal single-stranded DNA strands. The gel purification and following OD_260_ measurement suggested that with the nondiluted solid support, more than 40% of the products were obtained as the dimer and trimer (higher-mass oligomers are excluded here), and less than 60% of the starting strands were involved in the intrastrand coupling. By contrast, with a diluted strand, more than 95% was detected as the monomer that resulted from the intrastrand coupling.

This outcome provided further confirmation that (1) interstrand coupling indeed occurs in the on-solid support coupling reactions as a major conversion route, and (2) by increasing the distances between ODNs that possess reactive sites using the biotin dilution strategy, interstrand coupling was significantly suppressed.

These results hinted that longer coupling partners may favor interstrand coupling over intrastrand coupling. Instead of suppressing interstrand couplings, we could promote interstrand cross-linking between ss-DNAs and fabricate novel materials conjugated to multiple ssDNA. To enable these interstrand couplings, a new series of hexaarylbis(trimethylstannyl)benzenes were synthesized as coupling partners for Stille reactions. Precise mass determination using MALDI-TOF analysis for these high-mass species, particularly those with large aromatic moieties, proved challenging. However, PAGE analysis displayed characteristic bands indicating the formation of oligomers (Figure 6). The butylated bis(trimethylstannyl) coupling partner notably gave faint bands for oligomers, apparently with most of the material concentrated in the well. In contrast, the coupling partner lacking t-butyl groups exhibited a well-dispersed lane on the gel, showing intense bands corresponding to dimers and trimers. One possibility is that the hydrophobic nature of the *tert*-butyl modifications significantly reduces the solubility of DNA-polyaromatic conjugates in the aqueous solution. Moreover, the faint band for the monomer product in Lane 4 clearly indicated a suppression of the intrastrand coupling while interstrand coupling dominated, which agreed with our hypothesis.

**Figure 6:**
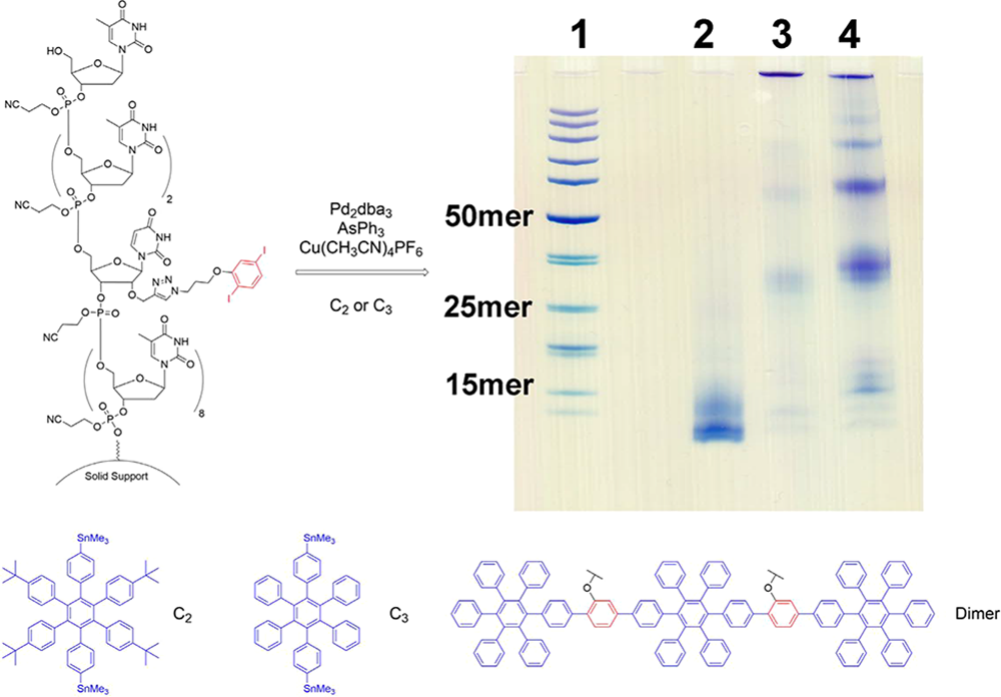
20% denaturing PAGE: Lane 1: standard markers. Lane 2: starting materials. Lane 3: coupling products with C_2_. Lane 4: coupling products with C_3_.

In summary, our strategy differentiates intra- and interstrand DNA cross-linking on solid support. We optimized solid phase Stille coupling of aryl iodides and bis(trimethylstannyl) coupling partners, using various ss-DNA containing all the canonical bases. The efficiency of our coupling reactions may be attributed to harsher reaction conditions and less hindrance of ssDNA compared to dsDNA coupling.^16,17^ Diluting the solid supported ODNs with capping reagents and reducing the proximity of reactive ssDNA maximized the yield of intrastrand coupling. Conversely, the use of large stannyl coupling partners promoted interstrand coupling. Intra- vs interstrand coupling products were easily characterized and purified by PAGE. The interstrand coupling reactions with large coupling partners creates possibilities for more intricate cross-links and fabrication of prototype PPP hybrid materials. Interstrand cross-linked DNA, which prevents DNA separation during transcription and replication, is useful in its own right.^53^ Pristine DNA-PPP ladder products were freely soluble in water and owed to the high compression strength of PPP (207 MPa),^38^ there’s potential applications in DNA origami to improve the structural integrity of DNA nanoconstructs. Further, using DNA self-assembly, the DNA-PPP would allow directional orientation of monodisperse PPP^54^ - a long-standing challenge - to provide a pathway to produce better performing PPP based electronics (FET, Blue-OLED, sensors).^55,56^ These achievements in intra- and interstrand couplings present a convenient and controllable approach for DNA functionalization and the fabrication of PPP materials within DNA scaffolds.

## Supporting Information Available

Synthetic protocols, additional mass spectra, and other characterization data (PDF)
