# Supplementary material for: Control of Solid-Supported Intra- vs Interstrand Stille Coupling Reactions for Synthesis of DNA–Oligophenylene Conjugates
Source: Bioconjug Chem. 2024 Jul 24;35(8):1166–71. doi: 10.1021/acs.bioconjchem.4c00310 (PMC11342295; doi:10.1021/acs.bioconjchem.4c00310)
Supplement: Supplementary file 1 — bc4c00310_si_001.pdf [file bc4c00310_si_001.pdf]

Supplementary Information

Control of solid-supported intra- vs. inter-strand Stille coupling reactions for synthesis of DNA oligophenylene conjugates

Chu-Fan Yang, Thanuka Udumulla, Ruojie Sha*, James W. Canary*

Department of Chemistry, New York University, New York, NY 10003, USA.

*Corresponding Author. Email: james.canary@nyu.edu; ruojie.sha@nyu.edu

Table of Contents

1. General Procedures --2
2. Synthesis of Azide Monomers ---3
3. Synthesis of Bis(trialkyltin) Coupling Partners ------------------------------------5
4. MALDI-TOF Characterization of Click Reactions---------------------------------6
5. Screening of Stille Reactions --12
6. MALDI-TOF Characterization of Stille Reactions ------------------------------13
7. MALDI-TOF Spectrum of the Pentaphenyl Product ----------------------------16
8. Formation of pentaphenyl products --17
9. PAGE of Stille products of heterobase strands -----------------------------------18
10. ^{1}H and ^{13}C NMR spectra---20
11. References ---28

General Procedures

Reagents for synthesis were purchased from Sigma-Aldrich and TCI America and used without further purification. ^{1}H NMR and ^{13}C NMR were collected using Bruker AV400 (400 MHz) NMR or Bruker AVIII600 (600 MHz) NMR. HR-MS was gathered using Agilent LCTOF (ESI source).

General procedure for click reactions. A 2.0 mL vial was charged with 5.0 mg of resin (20 nmol/mg DNA). A mixture of 4 µL of $CuSO_4$ (1 M in DI water), 4 µL of sodium ascorbate (2M in DI water), 80 µL of tris(benzyltriazolylmethyl)amine (TBTA, 0.1 M in 3:1 DMSO: *t*-butonal), and 200 µL of B_1 or B_2 (0.1 M in DMSO) was added and then heated at 60 °C for 16 h before dilution with 1 mL of acetone. After one-minute centrifugation, the supernate was decanted and the precipitate was washed with acetone twice, DI water twice, and acetone twice.

General procedure for Stille reactions of singly modified strands. A 2.0 mL vial was charged with 1.0 mg of resin, 0.9 mg of Pd_2dba_3 (1 µmol), 1.2 mg of $AsPh_3$ (4 µmol), 1.9 mg of trimethyl(phenyl)tin (8 µmol, 1.5 µL), and 300 µL of DMF. The mixture was heated at 80 °C for 10 h before dilution with 1 mL of acetone. Work-up was the same as that of click reactions.

General procedure for Stille reactions of doubly modified strands. A 2.0 mL vial was charged with 1.0 mg of resin, 18.2 µL of Pd_2dba_3 (0.01 g/mL in DMF), 4.8 µL of $AsPh_3$ (0.05 g/mL in DMF), 12.8 µL of 1, 4-bis(trimethylstannyl)benzene (0.05 g/mL in 1:1 DMF:Toluene), 30.0 µL of $Cu(CH_3CN)_4PF_6$ (0.01 g/mL in CH_3CN). The mixture was heated at 80 °C for 10 h before dilution with of 1 mL acetone. Work-up was the same as that of click reactions.

General procedure for Stille reactions with polyaromatic bistin coupling partner T_1 and T_2. A 2.0 mL vial was charged with 3.0 mg of resin, 27.3 µL of Pd_2dba_3 (0.01 g/mL in Toluene), 7,2 µL of $AsPh_3$ (0.05 g/mL in Toluene), polyaromatic bistin coupling partner $\mathbf{T_1}$ (0.2 µmol, 1.7 mg) or $\mathbf{T_2}$ (0.2 µmol, 2.2 mg), 22.5 µL of $Cu(CH_3CN)_4PF_6$ (0.02 g/mL in CH_3CN), 41.7 µL of Toluene. The mixture was heated at 80 °C for 10 h before dilution with 1 mL of acetone. Work-up was the same as that of click reactions.

Synthesis of oligo affinity support (OAS) bound DNA strands. OAS-bound DNA strands of desired sequences with 2' propargyl modification were synthesized in ABI 394 DNA Synthesizer by using natural DNA phosphoramidites (Glen Research), propargyl-modified phosphoramidite (Chemgenes) and oligo affinity support (Glen Research). A photocleavable linker (Glen Research) was used close to the 3' end to facilitate the cleavage of the strand from OAS.

Deprotection and cleavage of base-cleavable strands. To 1.0-5.0 mg base-cleavable strands was added 200 µL of 25% ammonium hydroxide. After 12 h, the mixture was diluted with 1.0 mL of DI water and centrifuged. The resin was discarded and the supernate was kept for further purification.

Deprotection and cleavage of photo-cleavable strands. To 1.0-5.0 mg photo-cleavable strands was added 200 µL of 25% ammonium hydroxide. After 12 h, the mixture was diluted with 1.0 mL DI water and centrifuged. The supernate was discarded and the resin was washed with DI water twice. 600 µL of NH_3/NH_4Cl buffer (pH=8.5) and 150 µL morpholine was added to the resin in a transparent vial. The mixture was exposed to 365 nm UV light (4 W) for 20 minutes. The resin was discarded and the supernate was kept for further purification.

Filtration of DNA solutions. To an Amicon Ultra-0.5 3K Centrifugal Filter Unit (MilliporeSigma) was added 500 μL of DNA solutions. The device was centrifuged at 14000 rcf for 10 minutes. 400 μL of NH_3/NH_4Cl buffer (pH=8.5) was added to the device and then centrifuged for additional 10 minutes to complete a buffer wash cycle. Repeated buffer washes for 3 times.

Denaturing Polyacrylamide Gel Electrophoresis. These gels contained 8.3 M urea and 20% acrylamide (19:1, acrylamide:bisacrylamide). The running buffer consisted of 100 mM Tris, 89 mM boric acid, 2 mM EDTA (1X TBE, pH 7.8). The sample buffer consisted of 10 mM NaOH, 1 mM EDTA, 90% formamide and 0.1% Xylene Cyanol FF tracking dye. Gels were run on a Hoefer SE-600 gel electrophoresis unit at 600 V at 55 °C. The gels were stained with ethidium bromide (EB) and imaged on Kodak Gel Logic 200 imaging System. GeneRuler Ultra-low range DNA ladder (Thermo Scientific) was used as the marker.

Nondenaturing Polyacrylamide Gel Electrophoresis. Gels contained 10% acrylamide (19:1, acrylamide:bisacrylamide). DNA was suspended in 10 μL of buffer containing 40 mM Tris, 20 mM acetic acid, 2 mM EDTA, and 12.5 mM magnesium acetate (1X TAE/Mg, pH 7.8), and the solution was hybridized by slow cooling. Samples were then brought to a final volume of 10 μL and a concentration of 50 μM, with a solution containing 1X TAE/Mg, 50% glycerol, and 0.1% each of Bromophenol Blue and Xylene Cyanol FF tracking dyes. Gels were run on a Hoefer SE-600 gel electrophoresis unit at 180 V at 4 °C and stained with Stainsall dye.

Synthesis of Azide Monomers

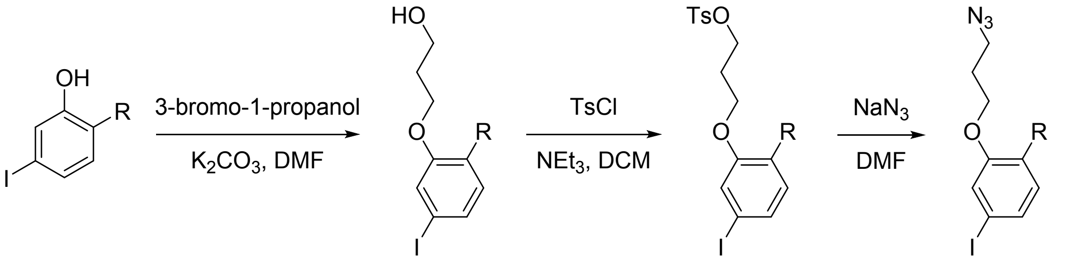

a_1: R = I, **b_1**: R = I, **c_1**: R = I, **B_1**: R = I,
a_2: R = H **b_2**: R = H **c_2**: R = H **B_2**: R = H

Figure S1. Synthesis of building blocks **S_1** and **S_2**.

Compound **b_1**: To a solution of **a_1** (2.87 g, 8 mmol) in DMF (15 mL) was added K_2CO_3 (5.5 g, 40 mmol) and 3-bromo-1-propanol (0.72 mL, 8 mmol), which was then stirred at 60 °C overnight. The suspension was cooled to room temperature before adding ethyl acetate (100 mL) and washing with water (50 mL × 4), brine (50 mL × 2). The organic layer was concentrated and purified by column chromatography to give **b_1** as a white solid. Yield: 2.2 g, 68 %. ^{1}H NMR (400 MHz, $CDCl_3$): δ (ppm) 7.45 (d, J = 8 Hz, 1H), 7.11 (s, 1H), 7.04 (d, J = 8 Hz, 1H), 4.15 (t, J = 6 Hz, 2H), 3.93 (t, J = 6 Hz, 2H), 2.14-2.08 (m, 2H), 1.85 (br, 1H). ^{13}C NMR (100 MHZ, $CDCl_3$): δ (ppm) 158.07, 140.56, 131.98, 121.38, 94.01, 86.30, 67.60, 60.55, 31.86. HRMS (ESI): Calcd for $C_9H_{10}I_2O_2$ (MNa^+): 426.8662; found: 426.8477.

Compound $\mathbf{c_1}$: A solution of $\mathbf{b_1}$ (2.2 g, 5.5 mmol) in DCM (25 mL) was cooled to 0 °C before adding triethylamine (1.53 mL, 11 mmol) and 4-toluenesulfonyl chloride (1.25 g, 6.5 mmol). The mixture was slowly warmed to room temperature and stirred overnight. The reaction mixture was diluted with 25 mL of DCM and washed with water (50 mL × 4), brine (50 mL × 2). The organic layer was concentrated and purified by column chromatography to give $\mathbf{c_1}$ as a white solid. Yield: 2.0 g, 66 %. ^{1}H NMR (400 MHz, $CDCl_3$): δ (ppm) 7.74 (d, J = 6.8 Hz, 2H), 7.28-7.24 (m, 2H), 7.10-7.08 (m, 1H), 6.96 (t, J = 6.4 Hz, 1H), 6.73 (dd, J_1 = 6.4 Hz, J_2 = 2 Hz, 1H), 4.22 (t, J = 5 Hz, 2H), 3.90 (t, J = 5 Hz, 2H), 2.39 (s, 3H), 2.12-2.07 (m, 2H). ^{13}C NMR (100 MHZ, CD_2Cl_2): δ (ppm) 158.05, 145.58, 140.90, 133.15, 132.32, 130.43, 128.28, 121.57, 94.21, 86.54, 67.49, 64.96, 29.18, 22.00. HRMS (ESI): Calcd for $C_{16}H_{16}I_2O_4S$ (MNa^+): 580.8751; found: 580.8494.

Compound $\mathbf{S_1}$: To a solution of $\mathbf{c_1}$ (2.0 g, 3.6 mmol) in DMF (15 mL) was added sodium azide (350 mg, 5.4 mmol). The mixture was stirred at room temperature before adding 100 mL of water and extracting with ethyl acetate (50 mL × 4). The organic layer was combined and washed with brine (50 mL × 4), dried over sodium sulfate, concentrated and purified by column chromatography to give $\mathbf{S_1}$ as a colorless liquid. Yield: 1.4 g, 90%. ^{1}H NMR (400 MHz, $CDCl_3$): δ (ppm) 7.45 (d, J = 8 Hz, 1H), 7.09 (d, J = 1.8 Hz, 1H), 7.05 (dd, J_1 = 8 Hz, J_2 = 2 Hz, 1H), 4.08 (t, J = 6 Hz, 2H), 3.62 (t, J = 6 Hz, 2H), 2.12-2.06 (m, 2H). ^{13}C NMR (100 MHZ, $CDCl_3$): δ (ppm) 157.88, 140.65, 132.08, 121.47, 93.95, 86.37, 66.05, 48.25, 28.75. IR ν_{max} (cm^{-1}): 2093 found for azide.

$\mathbf{B_2}$ was synthesized from $\mathbf{a_2}$ following the same route as $\mathbf{B_1}$.

Compound $\mathbf{b_2}$ (reported by literature[1]): white solid. Yield: 79 %. ^{1}H NMR (400 MHz, $CDCl_3$): δ (ppm) 7.29-7.26 (m, 2H), 6.99 (t, J = 8 Hz, 1H), 6.87 (dd, J_1 = 8 Hz, J_2 = 2 Hz, 1H), 4.09 (t, J = 8 Hz, 2H), 3.85 (t, J = 8 Hz, 2H), 2.06-2.00 (m, 2H), 1.75 (br, 1H). ^{13}C NMR (100 MHZ, $CDCl_3$): δ (ppm) 159.49, 130.93, 130.13, 123.81, 114.34, 94.50, 65.84, 60.31, 32.03.

Compound $\mathbf{c_2}$: white solid. Yield: 66 %. ^{1}H NMR (400 MHz, $CDCl_3$): δ (ppm) 7.75 (d, J = 8Hz, 2H), 7.29-7.24 (m, 3H), 7.10-7.08 (m, 1H), 6.96 (t, J = 8 Hz, 1H), 6.72 (dd, J_1 = 8 Hz, J_2 = 2.5 Hz, 1H), 4.22 (t, J = 8 Hz, 2H), 3.90 (t, J = 8 Hz, 2H), 2.39 (s, 3H), 2.13-2.07 (m, 2H). ^{13}C NMR (100 MHZ, $CDCl_3$): δ (ppm) 159.11, 145.02, 132.85, 130.86, 130.21, 129.97, 127.99, 123.80, 114.03, 94.41, 66.87, 63.25, 28.85, 21.85. HRMS (ESI): Calcd for $C_{16}H_{17}IO_4S$ (MNa^+): 454.9784; found: 454.9562.

Compound $\mathbf{B_2}$: colorless liquid. Yield: > 95 %. ^{1}H NMR (400 MHz, $CDCl_3$): δ (ppm) 7.29 (dt, J_1 = 8 Hz, J_2 = 1.2 Hz, 1H), 7.26-7.25 (m, 1H), 7.00 (t, J = 8 Hz, 1H), 6.86 (dd, J_1 = 8 Hz, J_2 = 2.5 Hz, 1H), 4.02 (t, J = 8 Hz, 2H), 3.51 (t, J = 8 Hz, 2H), 2.07-2.01 (m, 2H). ^{13}C NMR (100 MHZ, $CDCl_3$): δ (ppm) 159.35, 130.96, 130.24, 123.79, 114.30, 94.52, 64.81, 48.26, 28.82. IR ν_{max} (cm^{-1}): 2095 found for azide.

Synthesis of Bis(trialkyltin) Coupling Partners

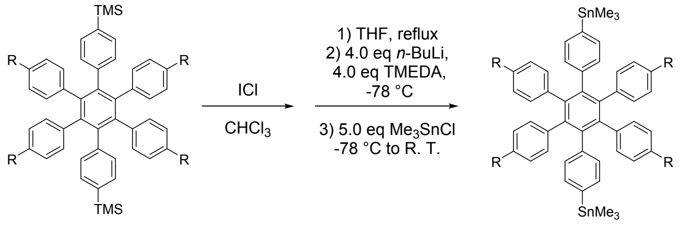

Figure S2. Synthesis of building blocks $\mathbf{T_1}$ and $\mathbf{T_2}$.

Compound $\mathbf{d_1}$ and $\mathbf{d_2}$ were synthesized according to reported methods.[2, 3]

Compound $\mathbf{T_1}$: A solution of $\mathbf{d_1}$ (785 mg, 0.87 mmol) in DCM (20 mL) was cooled to 0 °C. A solution of ICl (487 mg, 3.0 mmol) in 3 mL of DCM was added dropwise. The mixture was slowly warmed to room temperature and stirred overnight. The quantity 20 mL of saturated $Na_2S_2O_3$ solution was added to the reaction mixture and stirred until colorless. The resulting mixture was extracted with DCM (50 mL × 3), and washed with water (50 mL × 4), brine (50 mL × 2). The organic layer was then dried over Na_2SO_4, and concentrated to approximately 5 mL. Methanol (50 m) was added to precipitate a white solid that was collected by filtration and dried in vacuo overnight. The white solid was obtained with a near-quantitative yield and used directly for the next step. A Schlenk tube was charged with 303 mg (0.3 mmol) of the white solid and 15 mL of anhydrous THF. The mixture was heated at reflux until the solution became clear. The colorless solution was then cooled with acetone/dry ice to -78 °C. Tetramethylethylenediamine (1.2 mmol, 180 μL), and *n*-BuLi (0.75 mL, 1.6 M in hexanes) were added dropwise. The reaction mixture was stirred at -78 °C for 15 min Me_3SnCl (1.5 mL, 1 M in THF) was added dropwise, stirred at -78 °C for additional 30 min, and warmed to room temperature. After stirring overnight, 50 mL of water and 100 mL of DCM were added. The organic layer was washed with water (50 mL × 4), brine (50 mL × 2), dried with Na_2SO_4, and concentrated to approximately 5 mL. Methanol (50 mL) was added to precipitate a white solid that was collected by filtration. The crude product was used without further purification. The resulting bisstannyl complex is sensitive to heat and column chromatography, and difficult to purify. Slow diffusion of methanol into a DCM solution provided a sample of greater purity. Yield after crystallization: colorless crystalline solid, 101 mg, 31%. ^{1}H NMR (600 MHz, $CDCl_3$): δ (ppm) 6.92 (d, J = 8 Hz, 2H), 6.84-6.75 (m, 14 H), 6.69-6.64 (m, 8H), 1.10 (s, 36 H), 0.11 (s, 18 H). ^{13}C NMR (150 MHZ, $CDCl_3$): δ (ppm) 147.59, 141.03, 140.51, 138.00, 137.75, 133.79, 131.39, 131.23, 126.49, 123.26, 34.20, 31.34, -9.58.

Compound $\mathbf{T_2}$: colorless crystalline solid. Yield after crystallization: 25%. ^{1}H NMR (600 MHz, $CDCl_3$): δ (ppm) 6.96-6.76 (m, 28H), 0.18-0.08 (m, 18H). ^{13}C NMR (150 MHZ, $CDCl_3$): δ (ppm) 140.77, 140.46, 138.37, 134.04, 131.63, 131.57, 131.20, 126.71, 125.32, 125.20, -9.45.

MALDI-TOF Characterization of Click Reactions

Table S1. MALDI-TOF spectra of products of click reactions.

Entry	Resin types	Modification	Azide	Result
**1**	Base-cleavable	Singly	Azide **B**$_2$	Figure S3
**2**	Base-cleavable	Doubly	Azide **B**$_1$	Figure S4
**3**	Base-cleavable	Triply	Azide **B**$_1$	Figure S5
**4**	Photo-cleavable	Singly	Azide **B**$_1$	Figure S6
**5**	Photo-cleavable	Doubly	Azide **B**$_1$	Figure S7
**6**	Photo-cleavable	Triply	Azide **B**$_1$	Figure S8

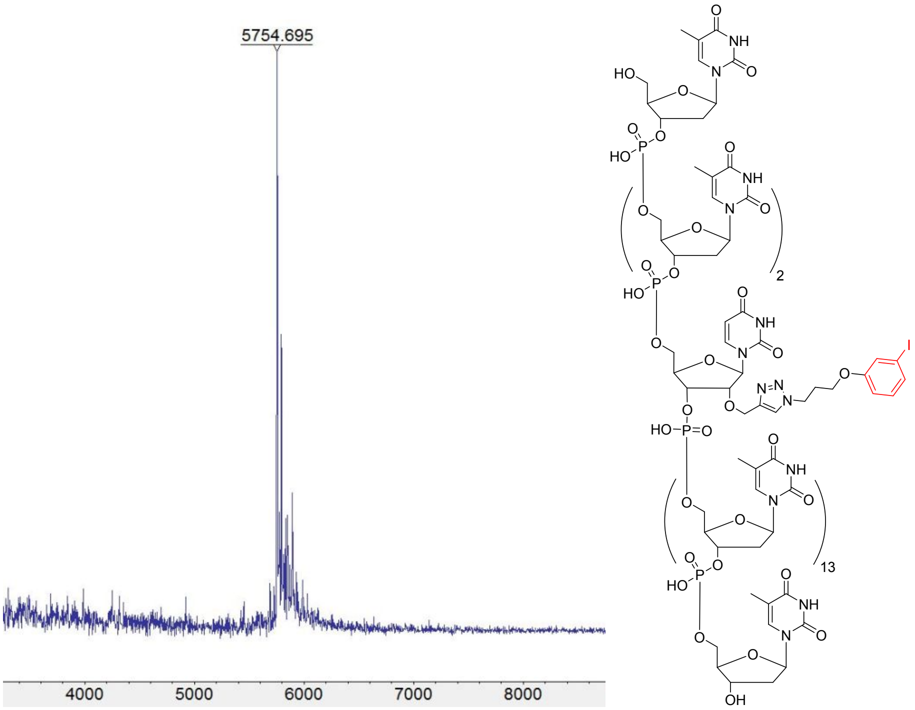

Figure S3. Base-cleavable ss-DNA-1: 5'-TTT M$_2$TT TTT TTT TTT TTT-3'. Calculated: 5755.6 ([M-H]$^-$). Found: 5754.7.

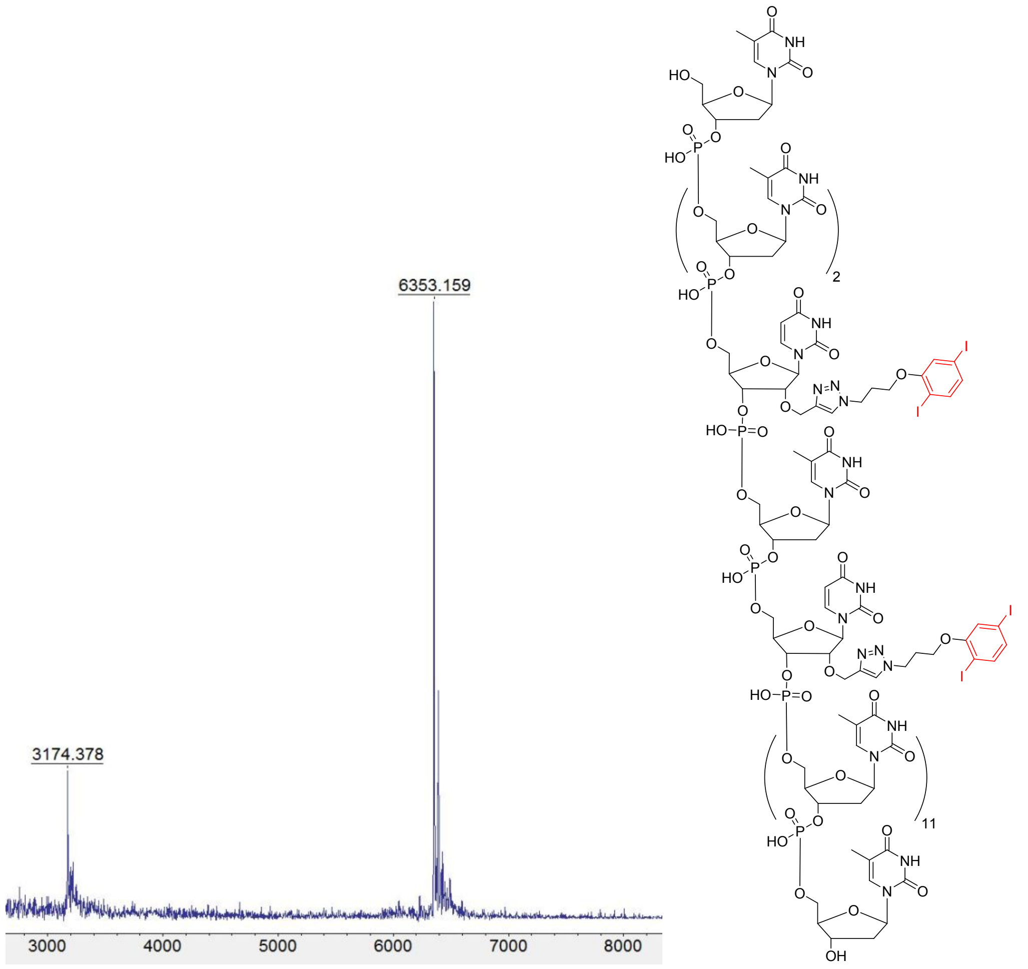

Figure S4. Base-cleavable ss-DNA-2: 5'-TTT M_1TM_1 TTT TTT TTT TTT-3'. Calculated: 6351.5 ($[M-H]^-$), 3174.8 ($[M-2H]^{2-}$). Found: 6353.1, 3174.4.

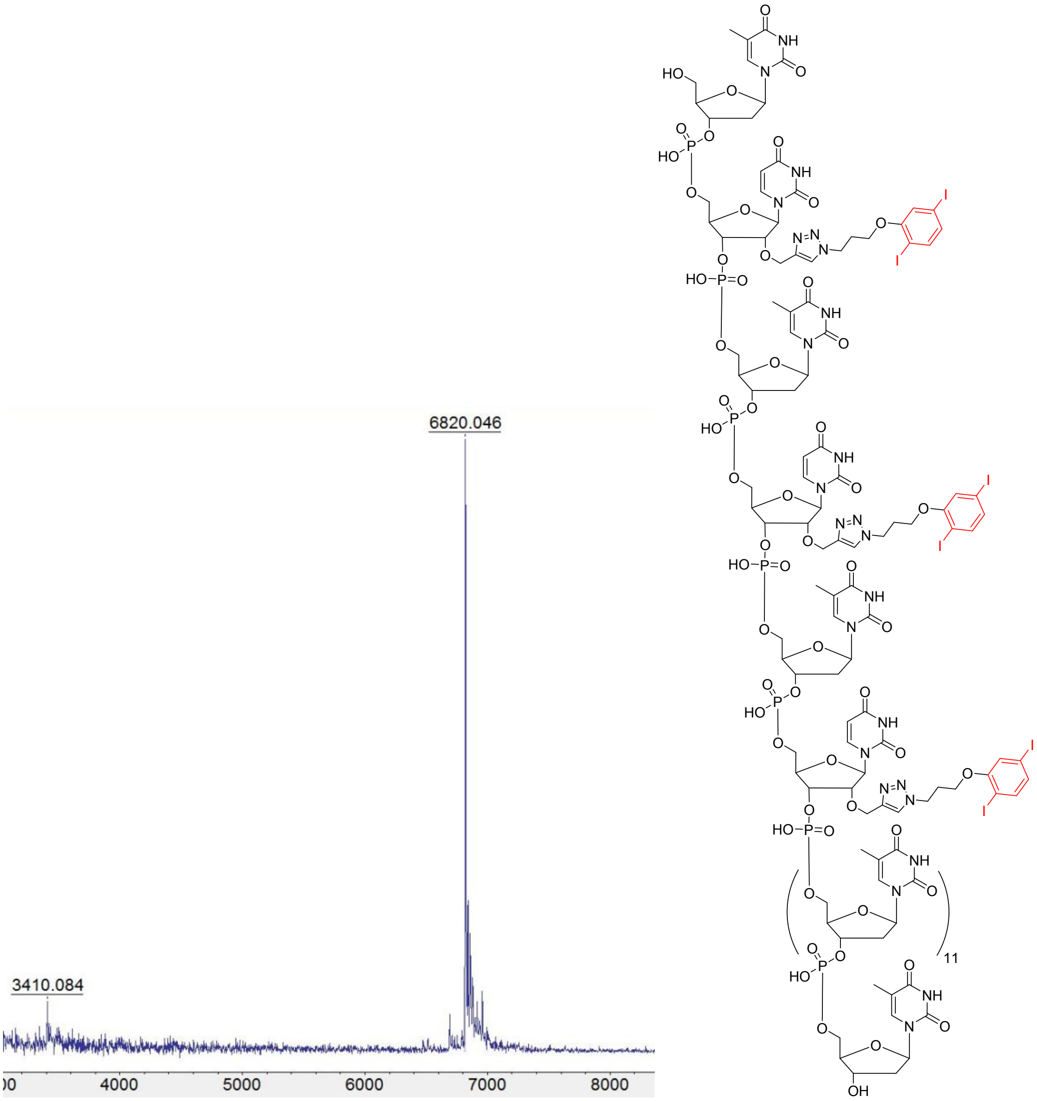

Figure S5. Base-cleavable ss-DNA-3: 5'-TM$_1$T M$_1$TM$_1$ TTT TTT TTT TTT-3'. Calculated: 6820.5 ([M-H]$^-$), 3409.8 ([M-2H]$^{2-}$). Found: 6820.0, 3410.1.

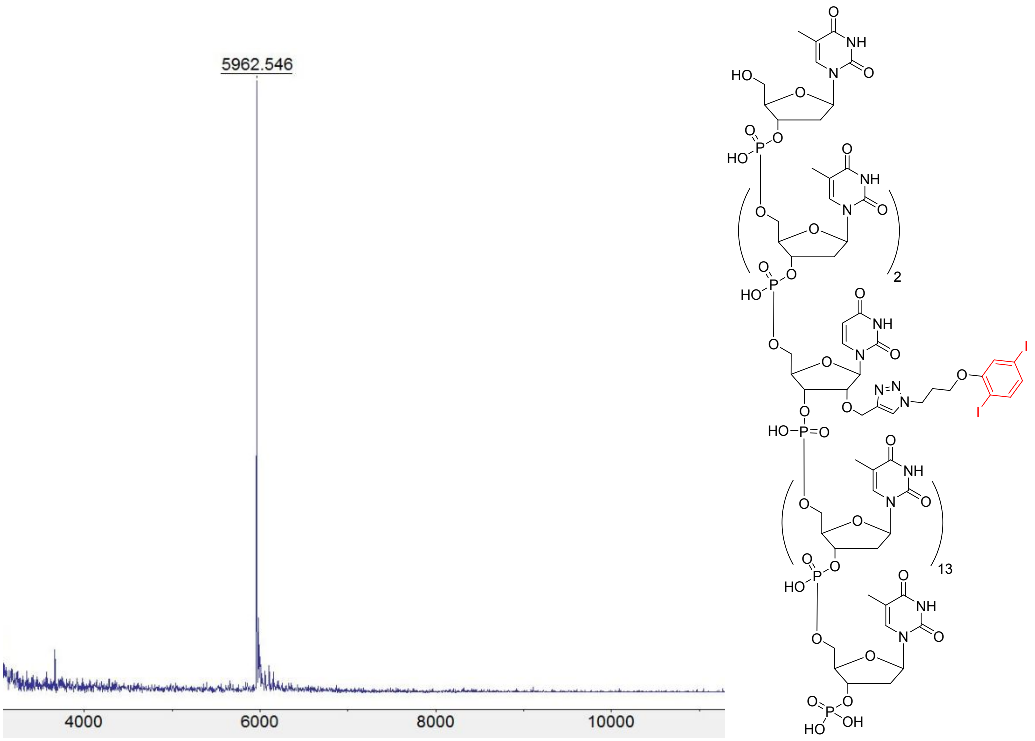

Figure S6. Photo-cleavable ss-DNA-1: 5’-TTT M_1TT TTT TTT TTT TTT-PC linker-TTT TTT-3’. Calculated: 5961.5 ($[M-H]^-$). Found: 5962.5.

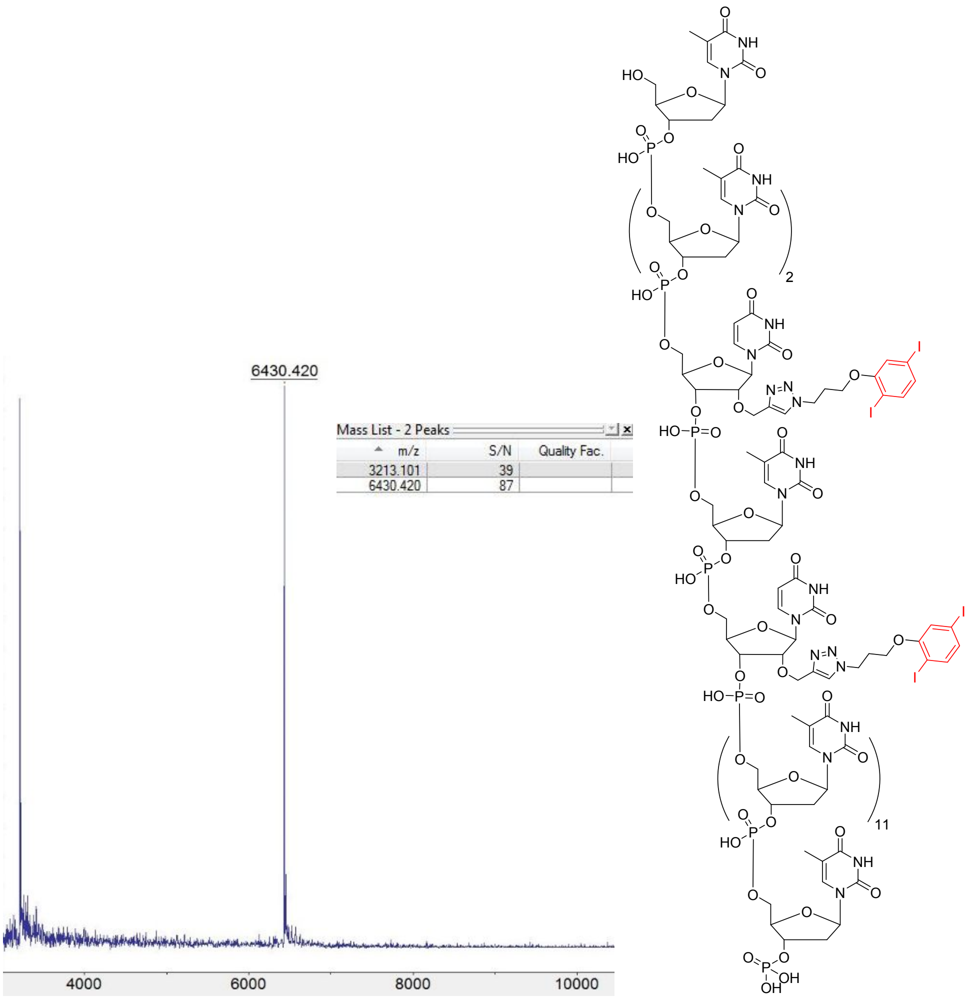

Figure S7. Photo-cleavable ss-DNA-2: 5'-TTT M_1TM_1 TTT TTT TTT TTT-PC linker-TTT TTT-3'. Calculated: 6430.5 ($[M-H]^-$), 3214.8 ($[M-2H]^{2-}$). Found: 6430.4, 3213.1.

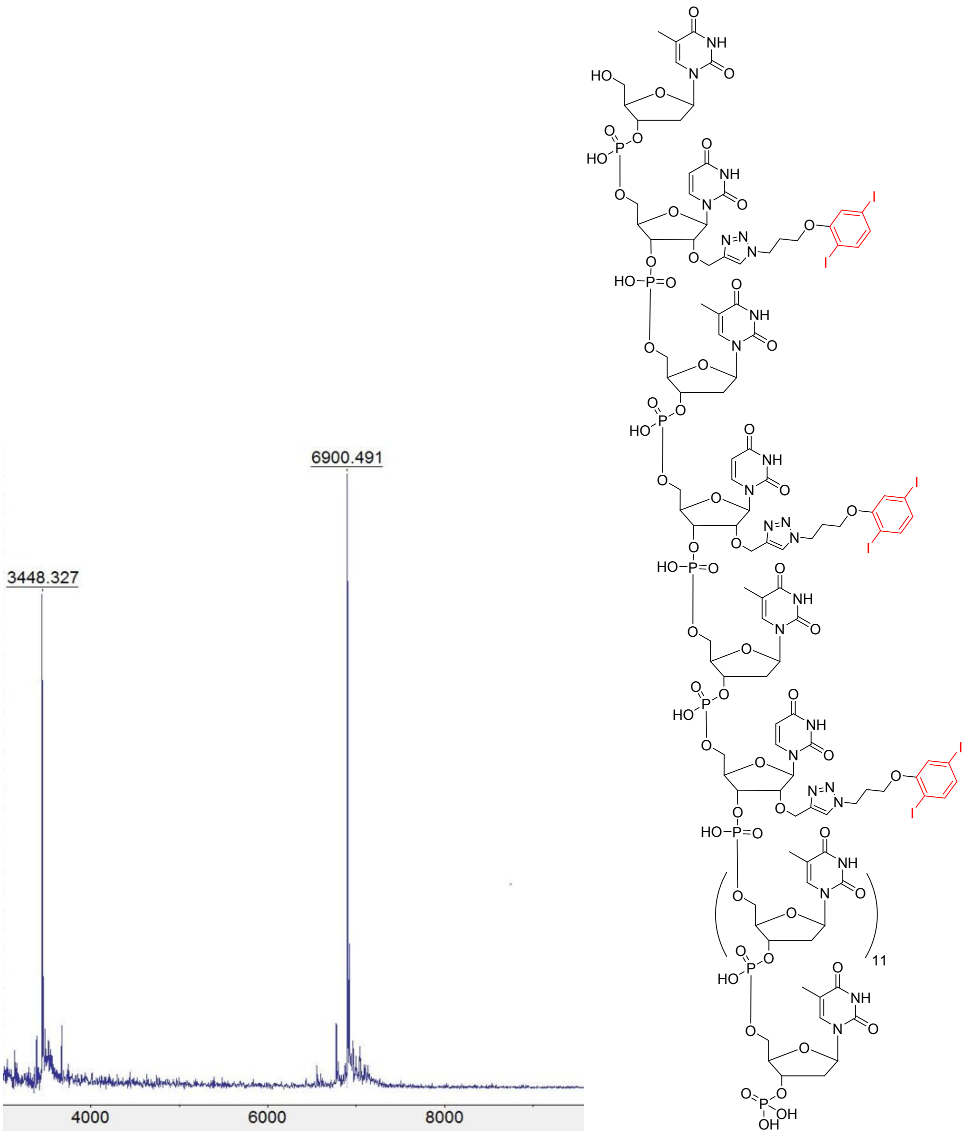

Figure S8. Photo-cleavable ss-DNA-3: 5'-TM_1T M_1TM_1 TTT TTT TTT TTT-PC linker-TTT TTT-3'. Calculated: 6899.5 ([M-H]$^-$), 3449.2 ([M-2H]$^{2-}$). Found: 6900.5, 3448.3.

Screening of Stille Reactions

Table S2. Screening the reaction of singly modified monoiodide ss-DNA (**1**) and phenyltrialkyltin.

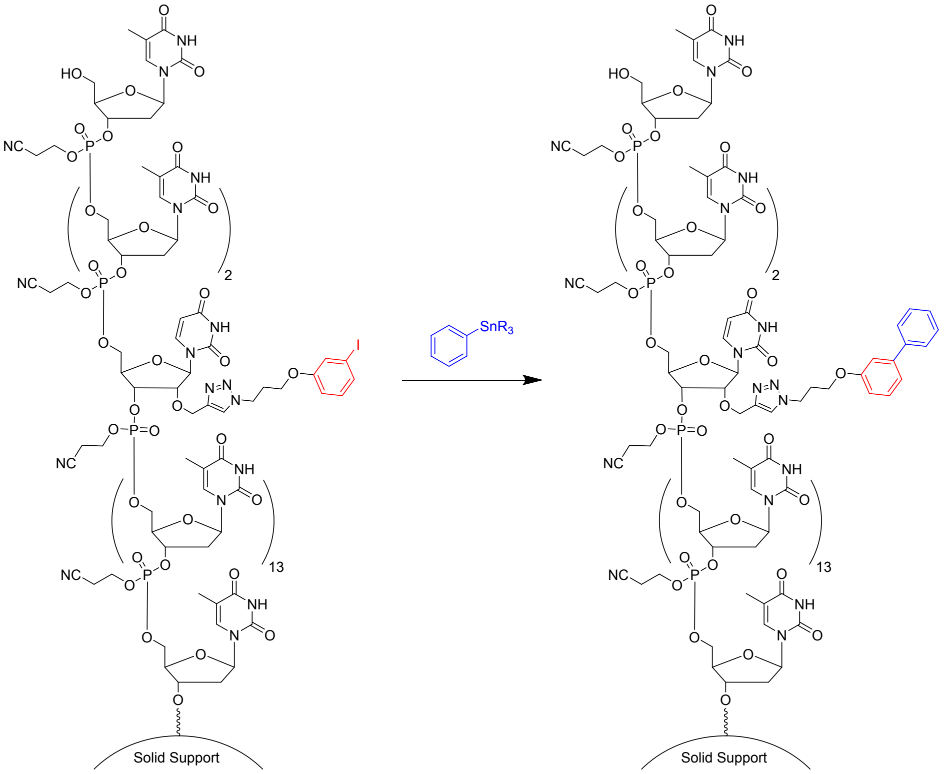

1 **2**

**Entry**	**Catalytic system**	**Additive**	**Tin**	**Temperature (°C)**	**Result**
**1**	$Pd_2dba_3/AsPh_3$	-	R = *n*-butyl	80	Figure S9
**2**	$Pd_2dba_3/AsPh_3$	-	R = methyl	80	Figure 10
**3**	$Pd_2dba_3/AsPh_3$	-	R = methyl	60	Figure S11
**4**	Pd_2dba_3	-	R = methyl	60	Unreacted
**5**	$Pd_2dba_3/AsPh_3$	CuI	R = methyl	60	Figure S12
**6**	$Pd_2dba_3/AsPh_3$	$Cu(CH_3CN)_4PF_6$	R = methyl	60	Figure S13
**7**	$Pd_2dba_3/AsPh_3$	$Cu(CH_3CN)_4PF_6$	R = methyl	80	Figure S14

MALDI-TOF Characterization of Stille Reactions

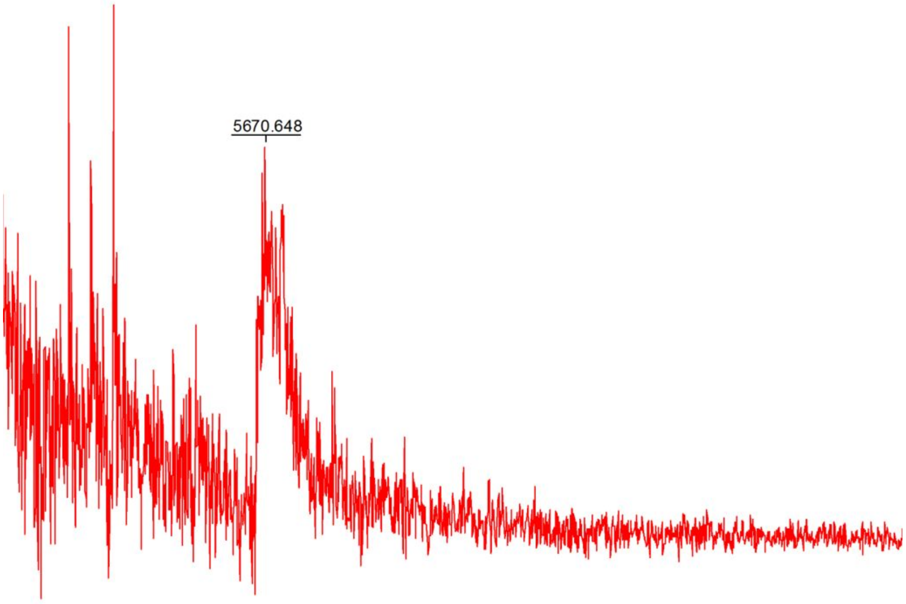

Figure S9. Stille coupling of base-cleavable ss-DNA-1 and phenyltributyltin. Calculated: 5705.8 ([M-H]$^-$), 2852.4 ([M-2H]$^{2-}$). Not found.

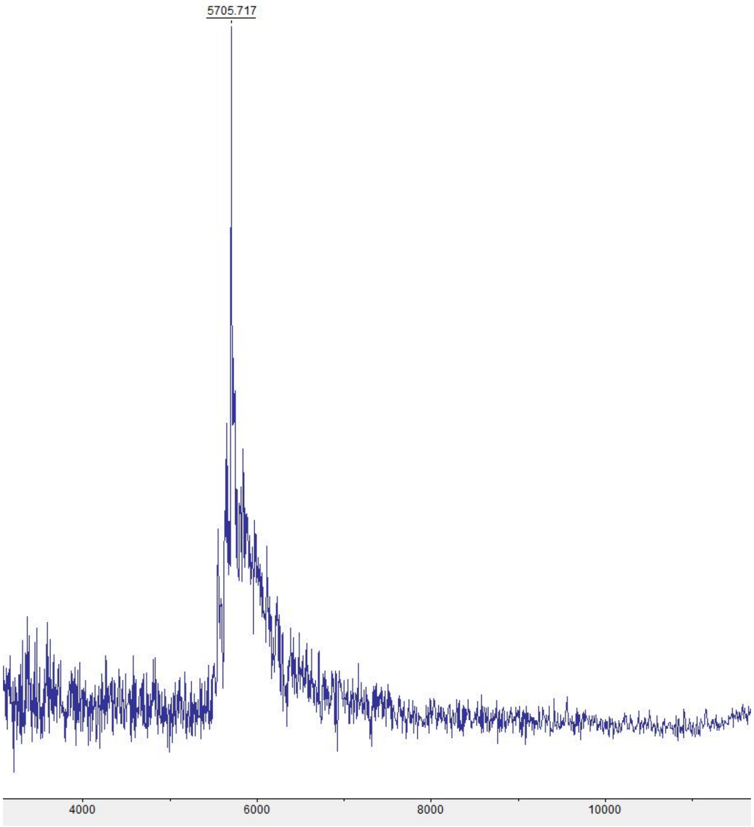

Figure S10. Stille coupling of base-cleavable ss-DNA-1 and phenyltrimethyltin at 80 °C. Calculated: 5705.8 ([M-H]$^-$). Found: 5705.7.

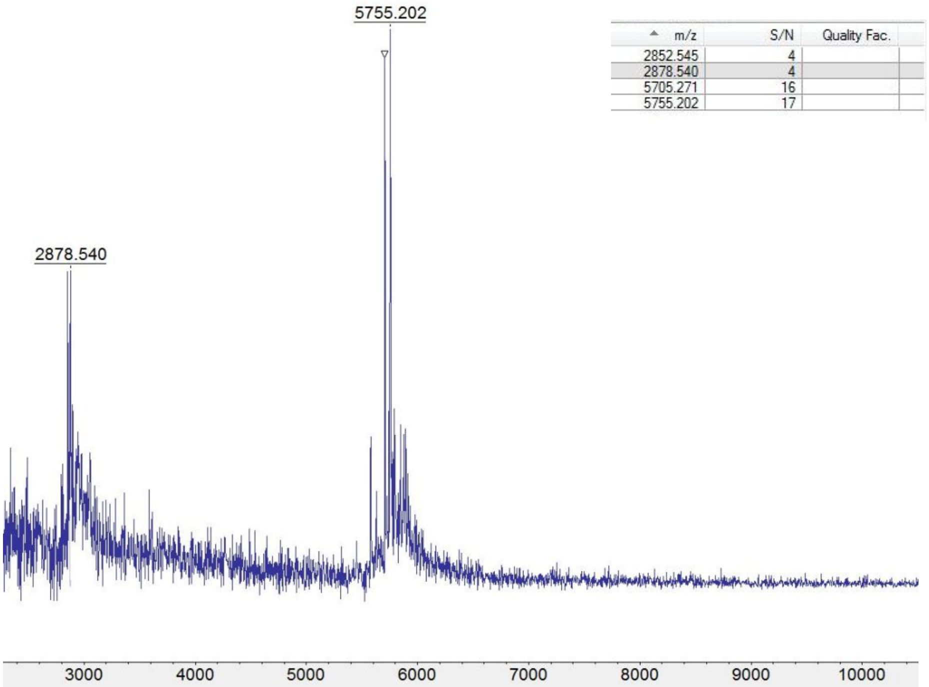

Figure S11. Stille coupling of base-cleavable ss-DNA-1 and phenyltrimethyltin at 60 °C. Calculated: 5705.8 ($[M-H]^-$), 2852.4 ($[M-2H]^{2-}$) for the product; 5755.6 ($[M-H]^-$), 2877.4 ($[M-2H]^{2-}$) for the starting material. Found: 5705.3, 2852.5 for the product; 5755.2, 2878.5 for the starting material.

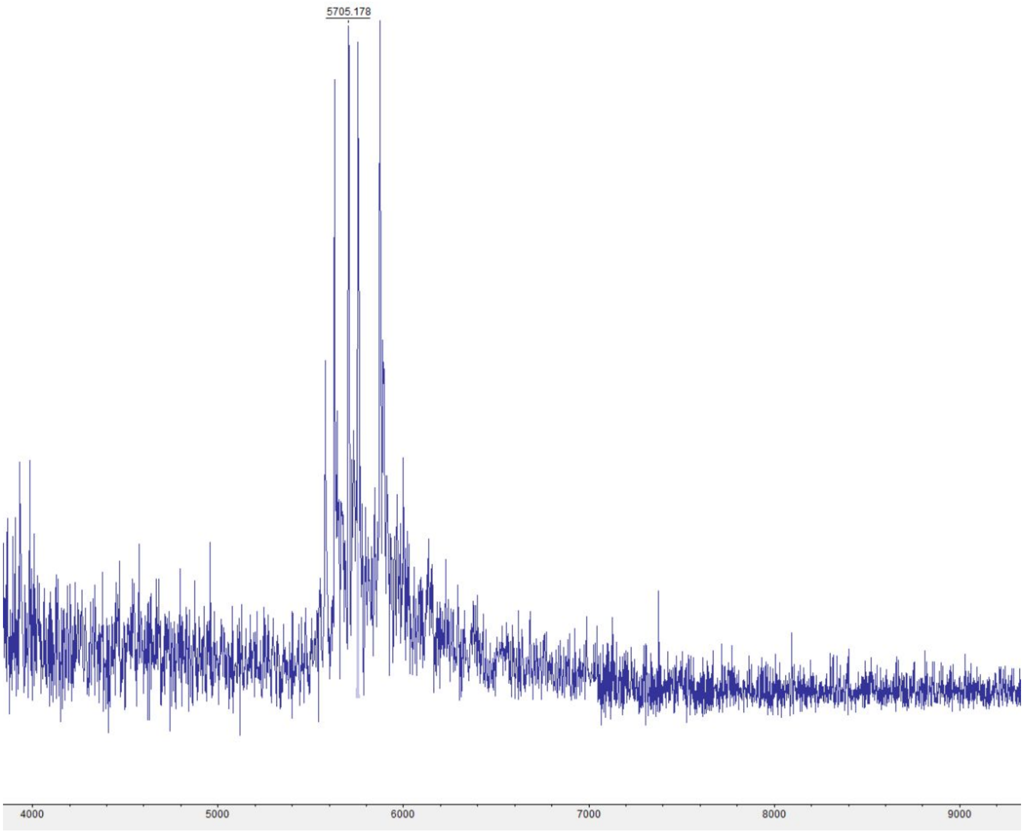

Figure S12. Stille coupling of base-cleavable ss-DNA-1 and phenyltrimethyltin at 60 °C in the presence of CuI. Calculated: 5705.8 ($[M-H]^-$) for the product, 5755.6 ($[M-H]^-$) for the starting material. Found: 5705.2 for the product, 5755.8 for the starting material.

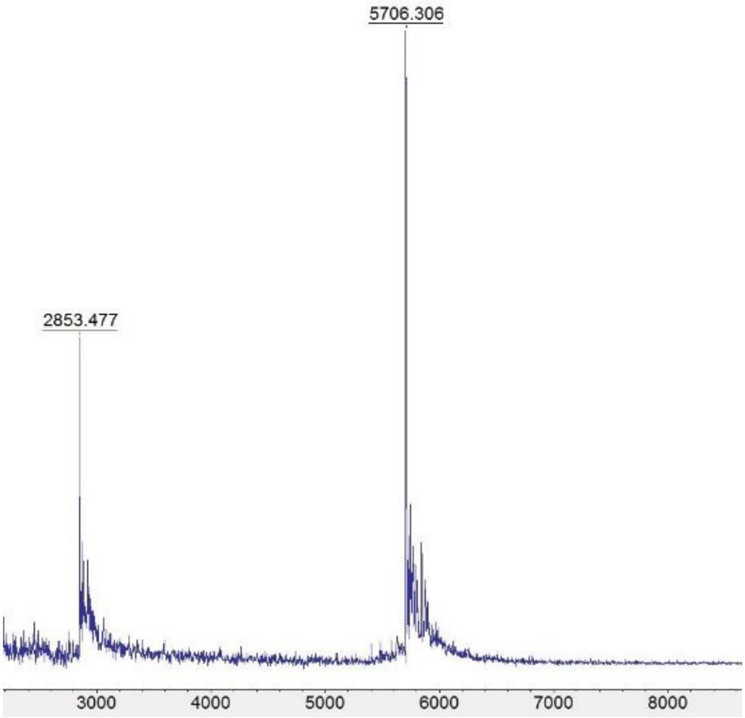

Figure S13. Stille coupling of base-cleavable ss-DNA-1 and phenyltrimethyltin at 60 °C in the presence of $Cu(CH_3CN)_4PF_6$. Calculated: 5705.8 ($[M-H]^-$), 2852.4 ($[M-2H]^{2-}$). Found: 5706.3, 2853.5.

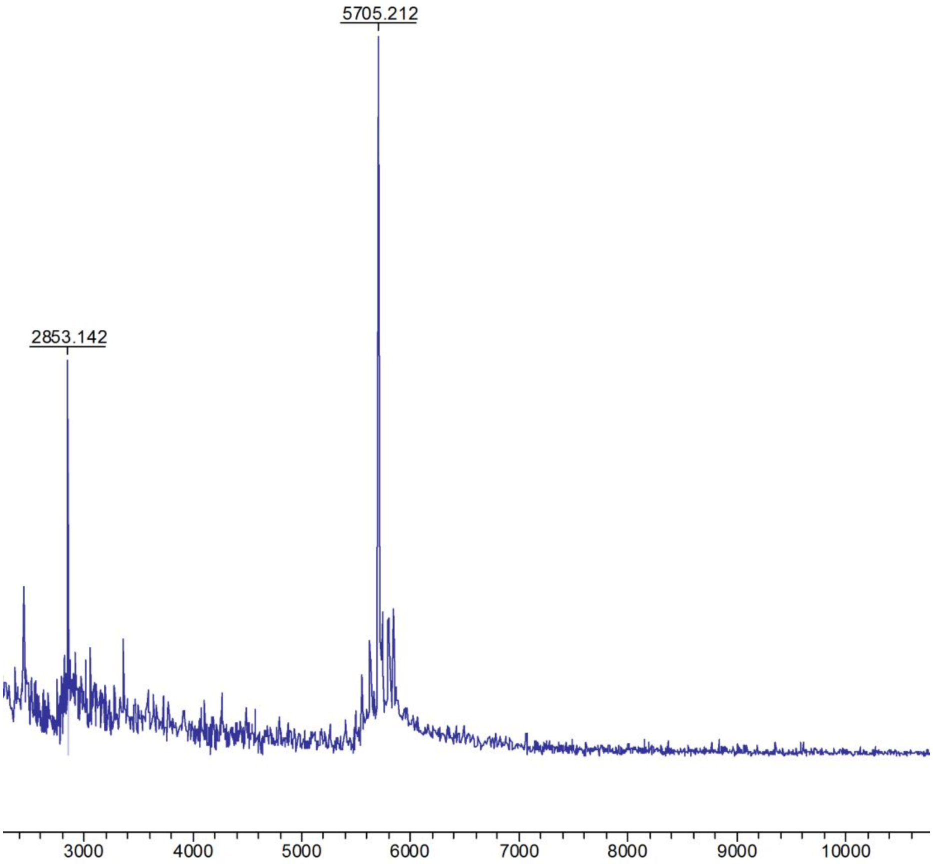

Figure S14. Stille coupling of base-cleavable ss-DNA-1 and phenyltrimethyltin at 80 °C in the presence of $Cu(CH_3CN)_4PF_6$. Calculated: 5705.8 ($[M-H]^-$), 2852.4 ($[M-2H]^{2-}$). Found: 5705.2, 2853.1.

MALDI-TOF Spectrum of the Pentaphenyl Product

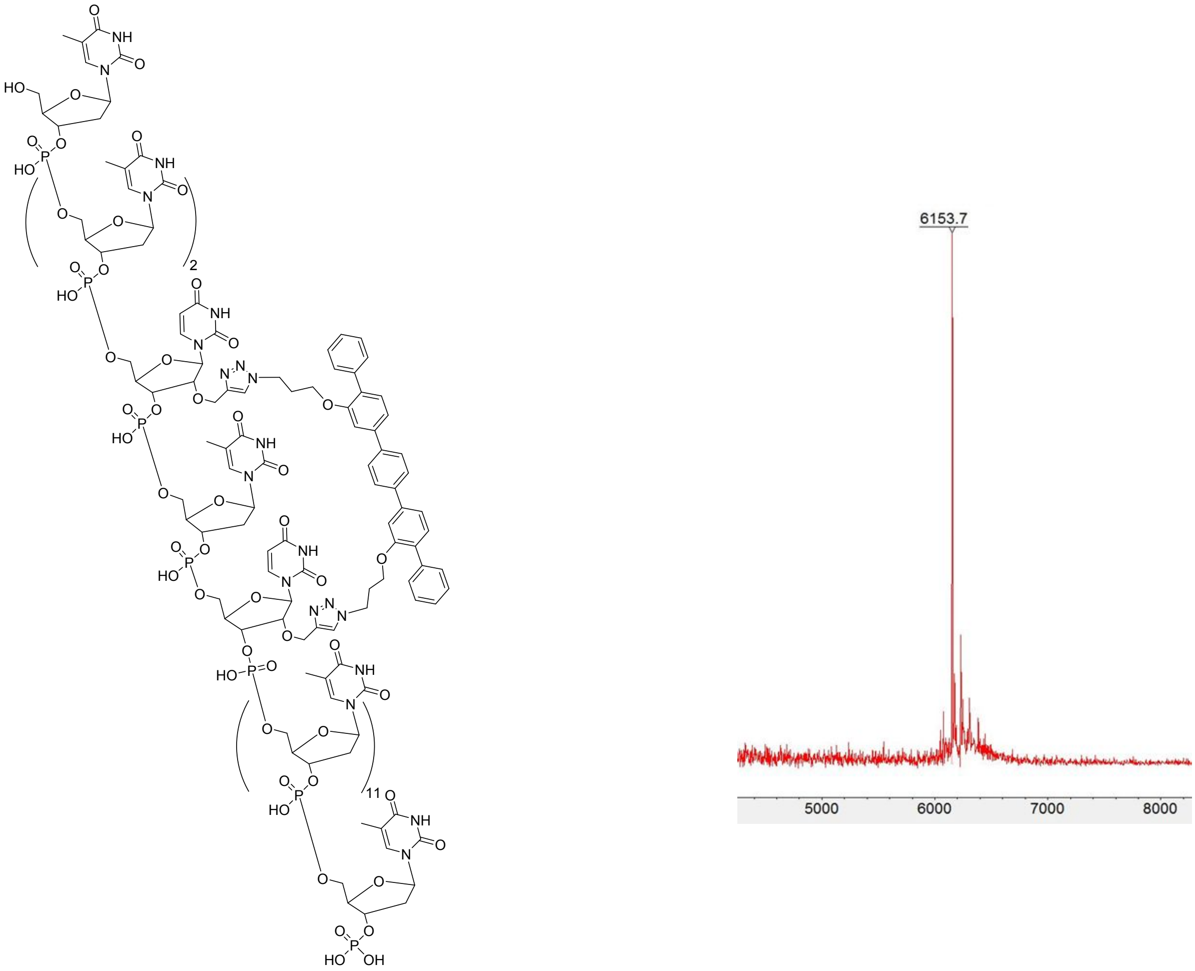

Figure S15. MALDI-TOF analysis of the intra-strand coupling product. Calculated: 6153.2 ([M-H]-). Found: 6153.7. Photo-cleavable ss-DNA-2: 5’-TTT M_1TM_1 TTT TTT TTT TTT-PC linker-TTT TTT-3’.

Formation of pentaphenyl products

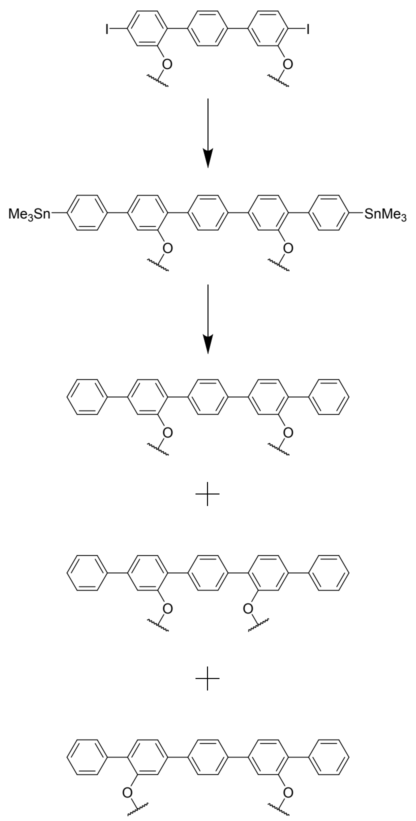

Figure S16. Formation and regioisomers of the pentaphenyl product.

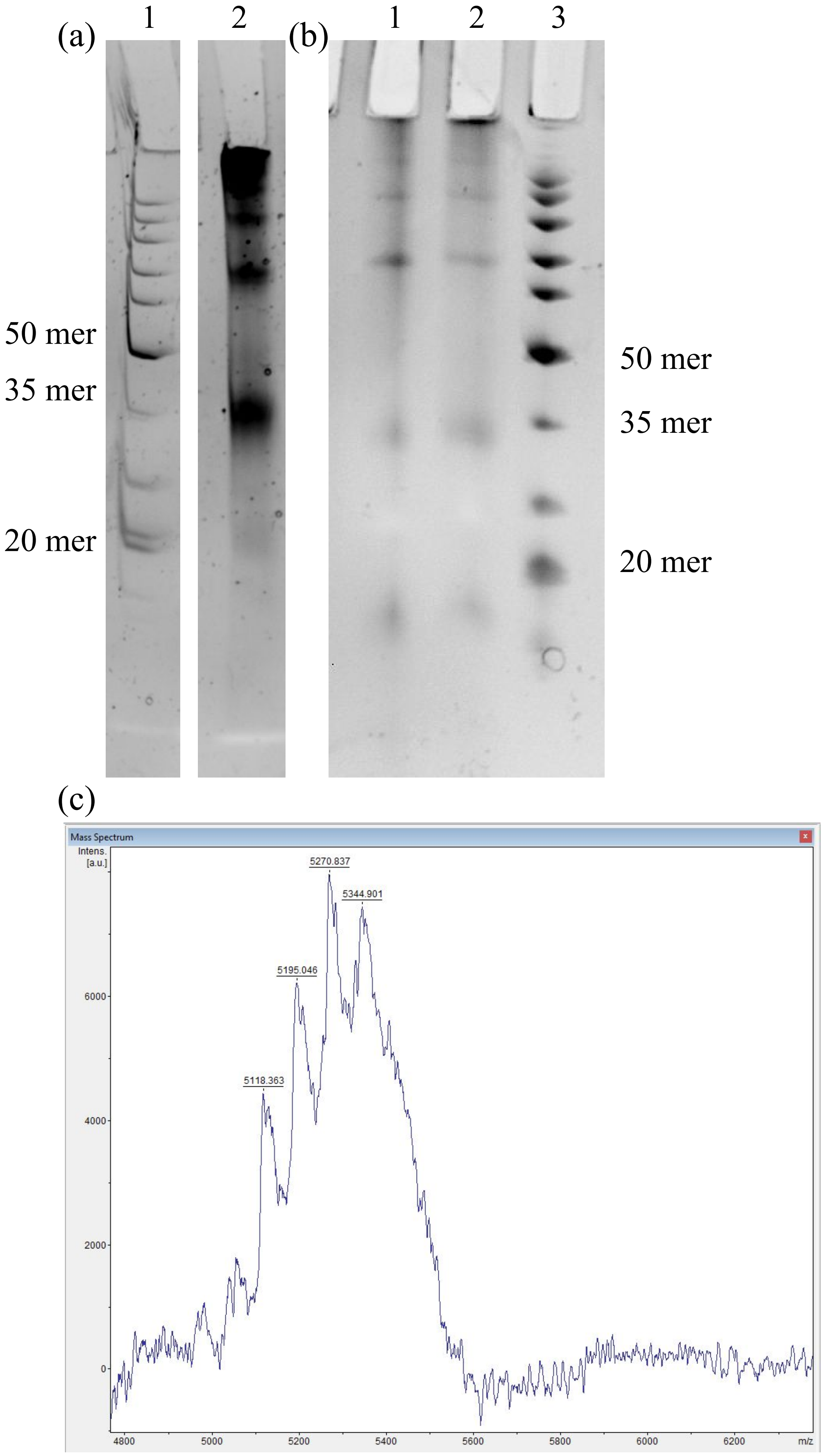
(a)
1
2
50 mer
35 mer
20 mer
(b)
1
2
3
50 mer
35 mer
20 mer
(c)
Mass Spectrum
Intens.
[a.u.]
5270.837
5344.901
5195.046
5118.363
6000
4000
2000
0
4800
5000
5200
5400
5600
5800
6000
6200
m/z

(d)

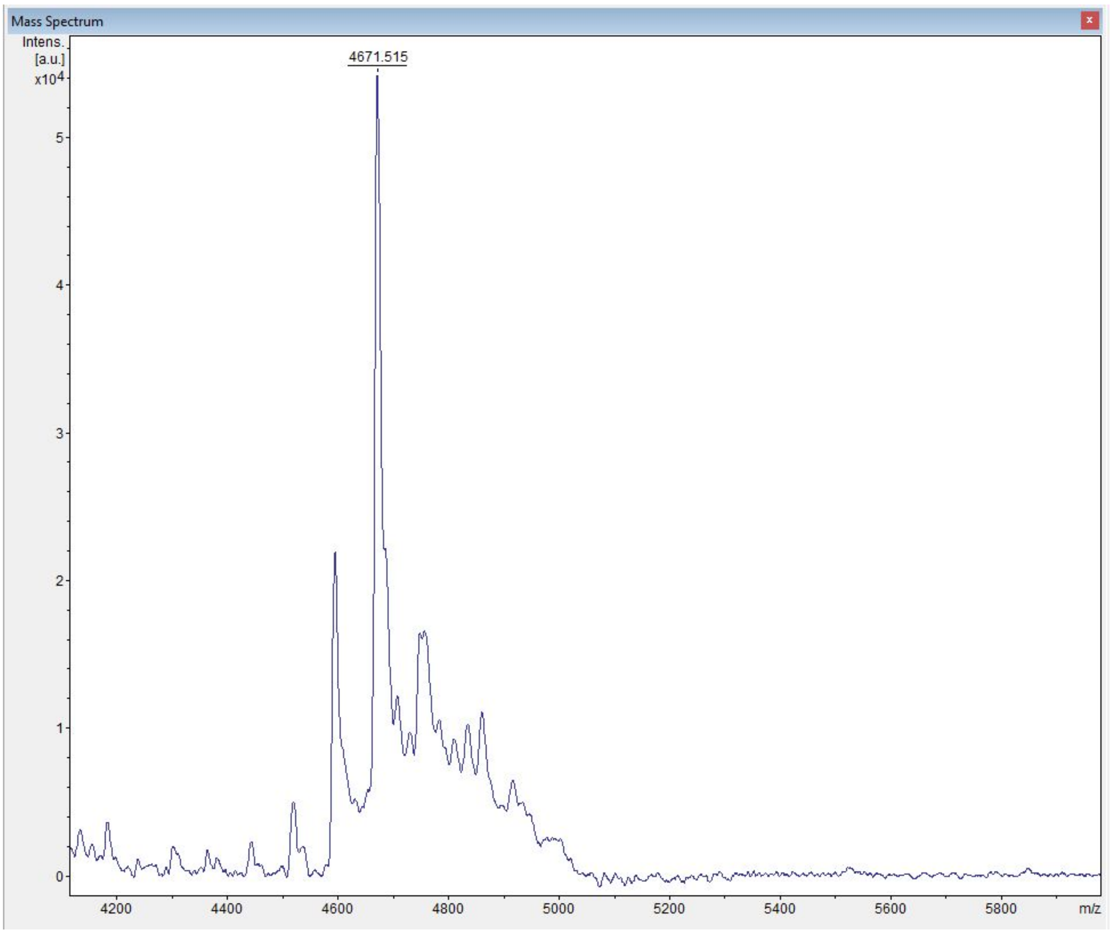

Figure S17. 20% denaturing PAGE with heterobase sequences. ss-DNA strands:
HB-S_1: 5'-M_1TG CAG TCT TTT TT-3'
HB-S_2: 5'- M_1TM_1 GCA GTC TTT TTT-3'
Coupling partner C1: 1, 4-bis(trimethylstannyl)benzene
(a) Lane 1: Standard marker. Lane 2: Stille products of HB-S_2 and C_1. (b) Lane 1 and 2: Stille products of HB-S_1 and C_1. Lane 3: Standard marker. (c) MALDI-TOF analysis of the reaction mixture of HB-S_2 and C_1. Calculated: 5271.4 ([M-H]$^-$). Found: 5270.8. (d) MALDI-TOF analysis of the reaction mixture of HB-S_1 and C_1. Calculated: 4674.0. ([M-H]$^-$). Found: 4671.5.

^{1}H and ^{13}C NMR spectra

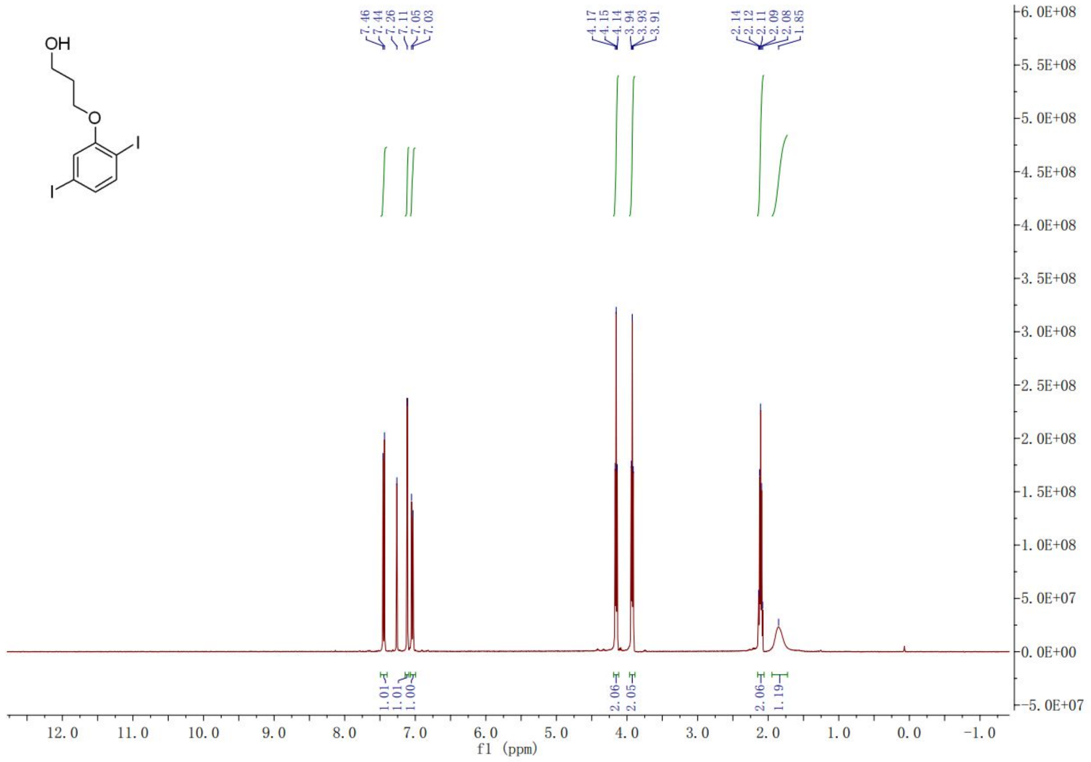

Figure S18. ^{1}H NMR of $\mathbf{b_1}$.

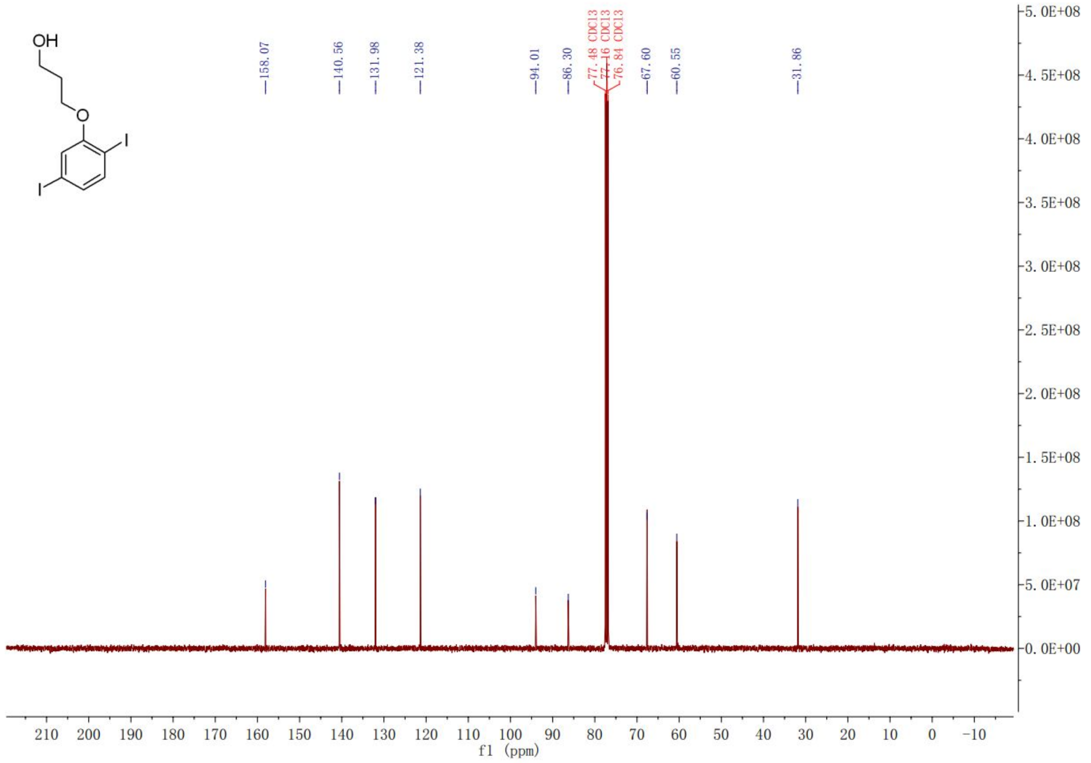

Figure S19. ^{13}C NMR of $\mathbf{b_1}$.

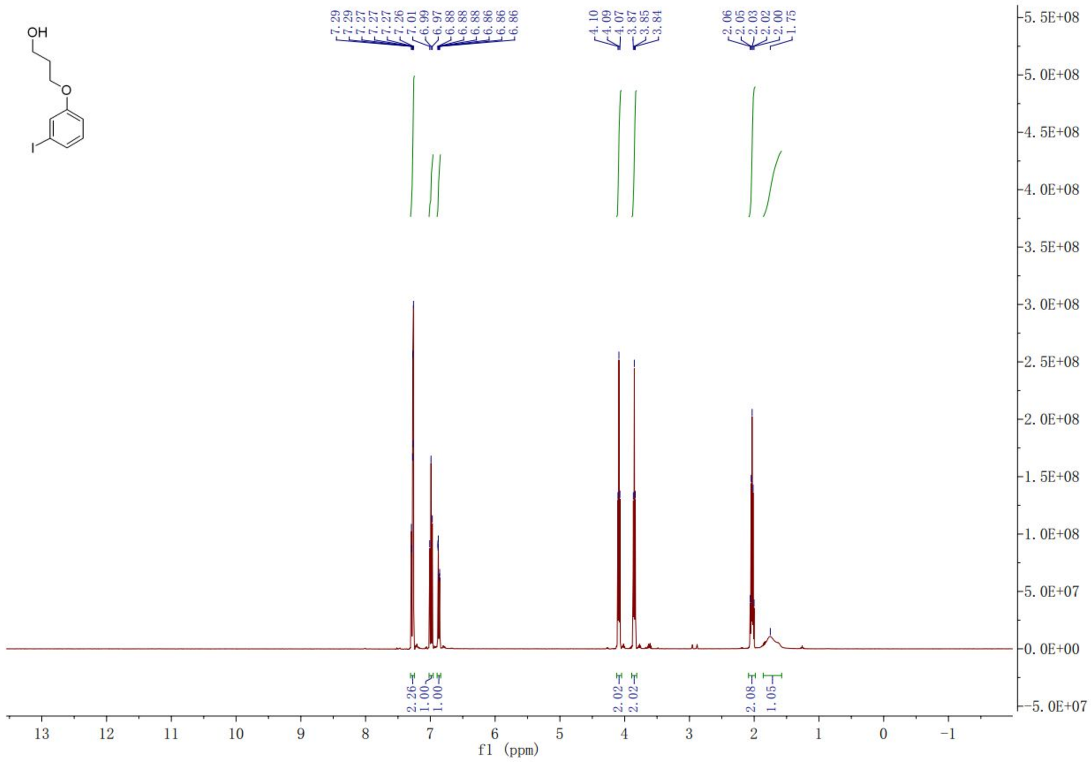

Figure S20. ^{1}H NMR of $\mathbf{b_2}$.

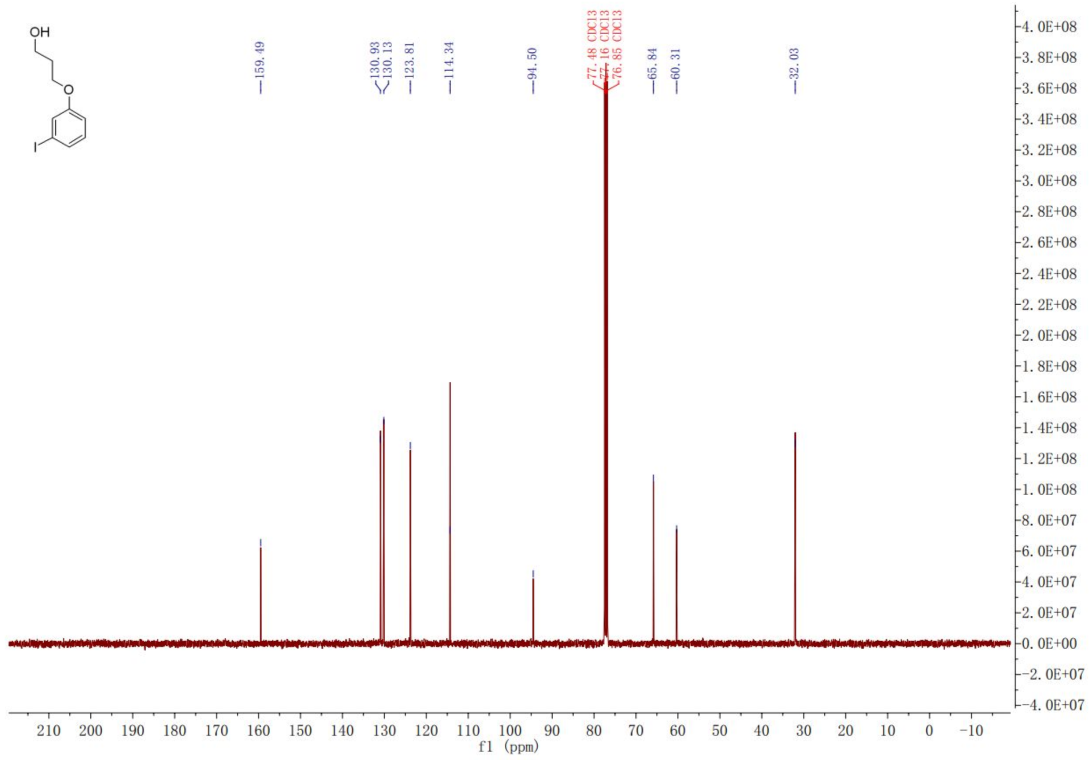

Figure S21. ^{13}C NMR of $\mathbf{b_2}$.

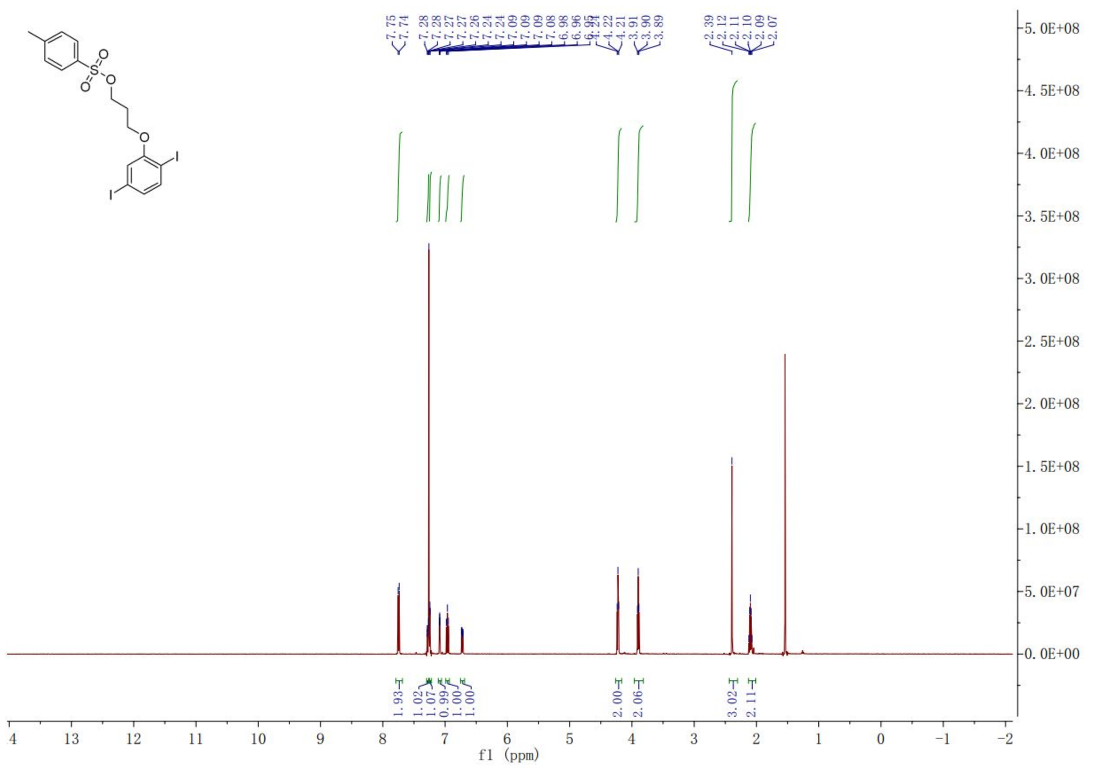

Figure S22. ^{1}H NMR of $\mathbf{c_1}$.

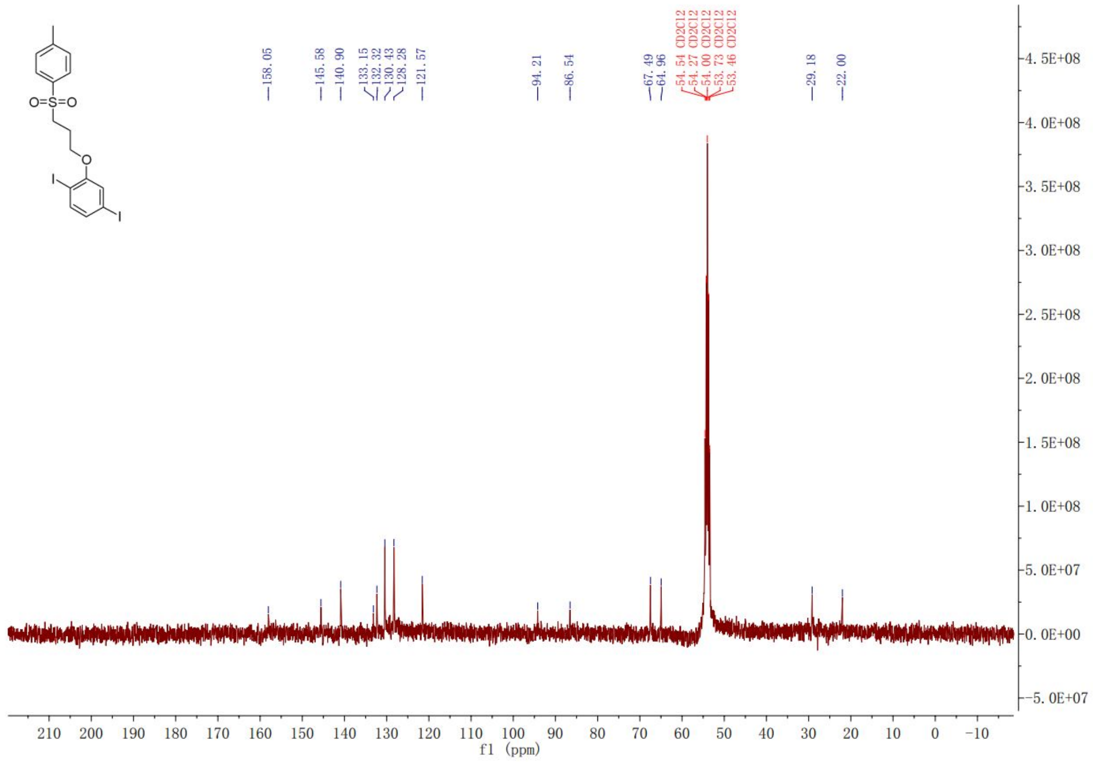

Figure S23. ^{13}C NMR of $\mathbf{c_1}$.

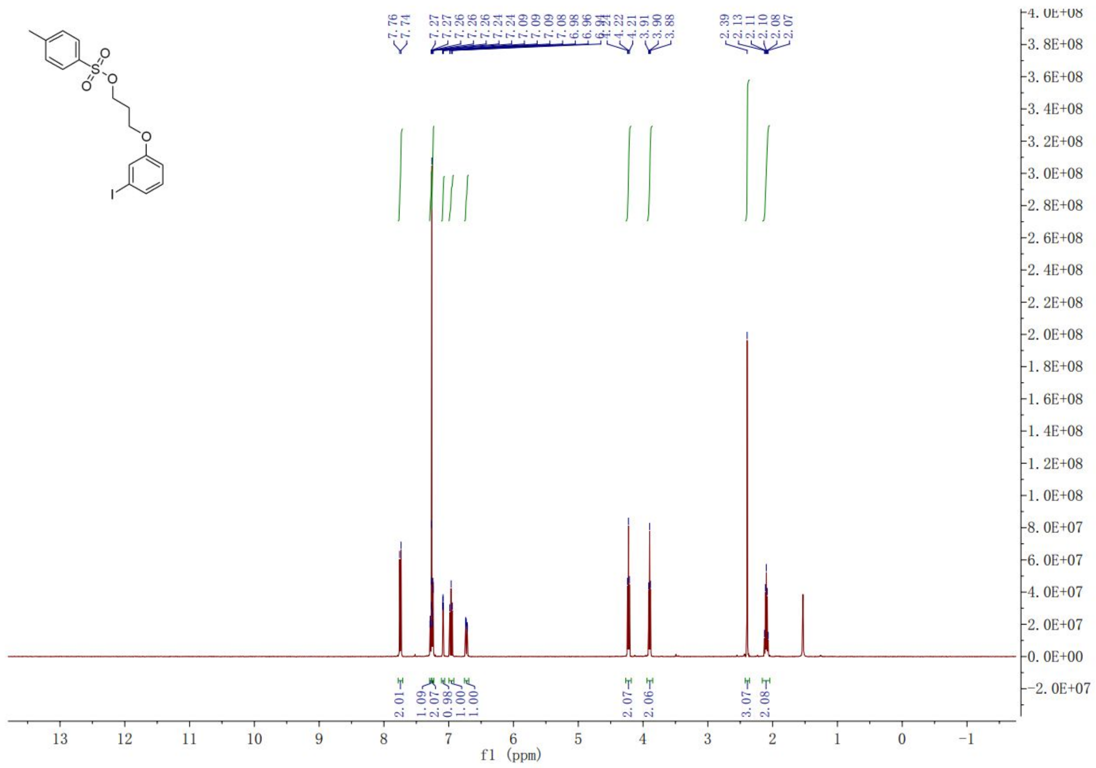

Figure S24. ^{1}H NMR of $\mathbf{c_2}$.

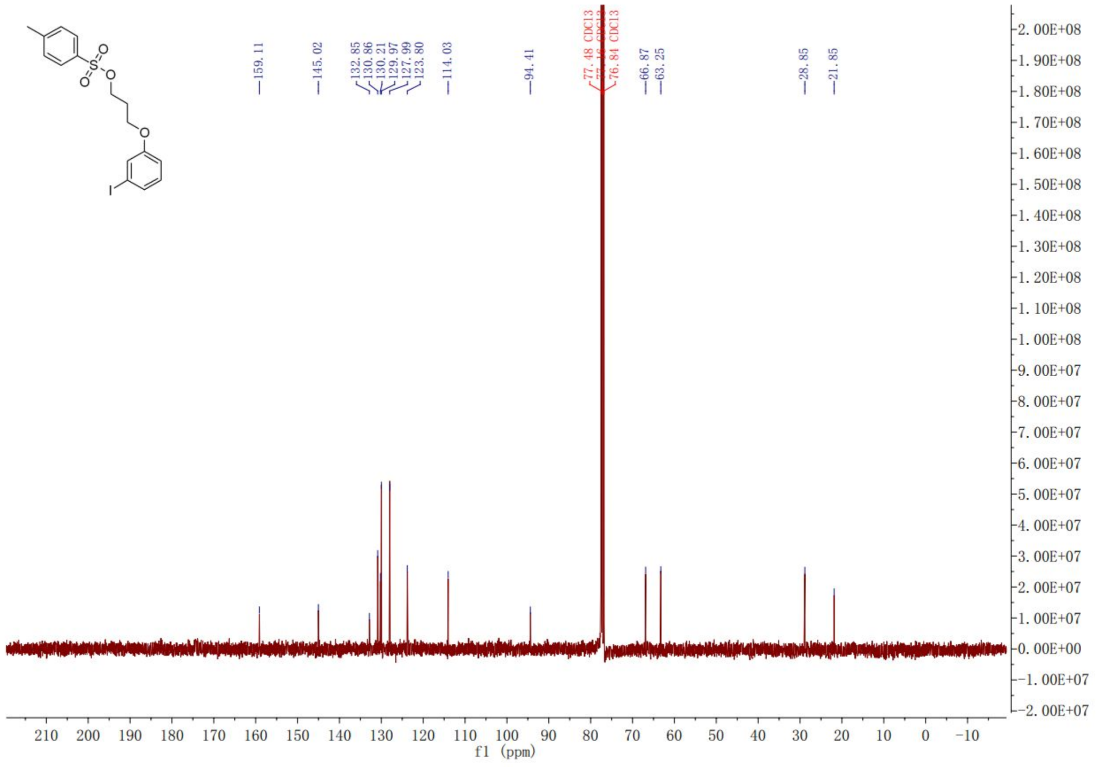

Figure S25. ^{13}C NMR of $\mathbf{c_2}$.

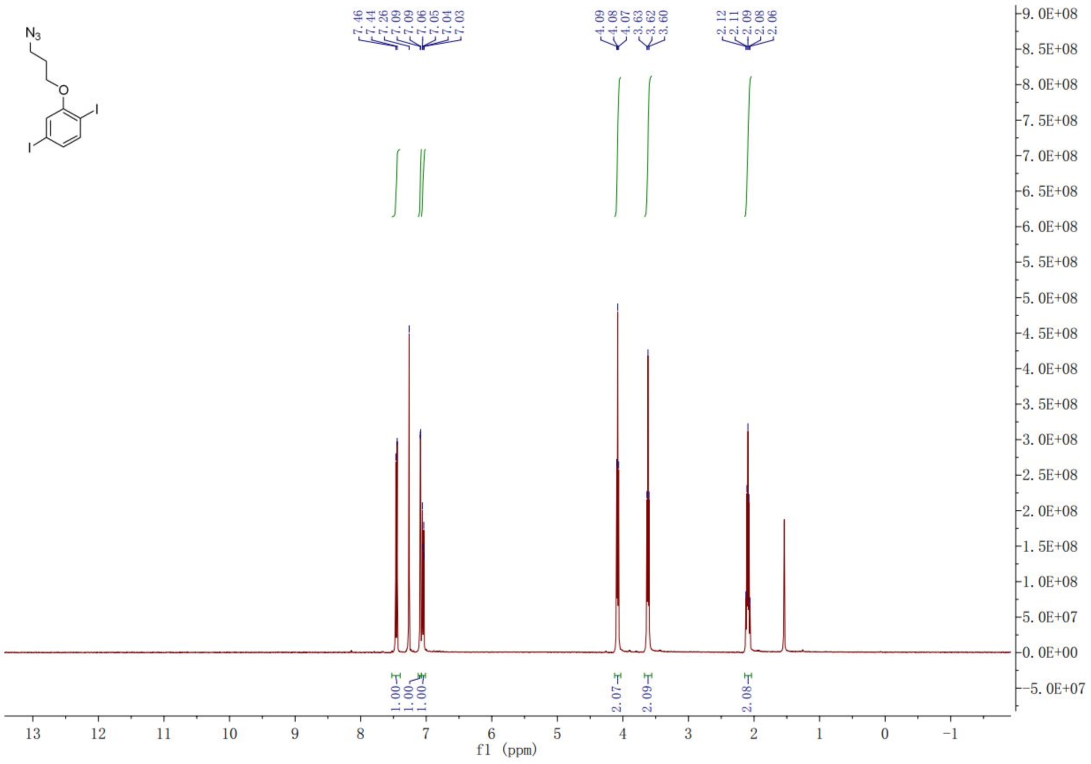

Figure S26. ^{1}H NMR of $\mathbf{S_1}$.

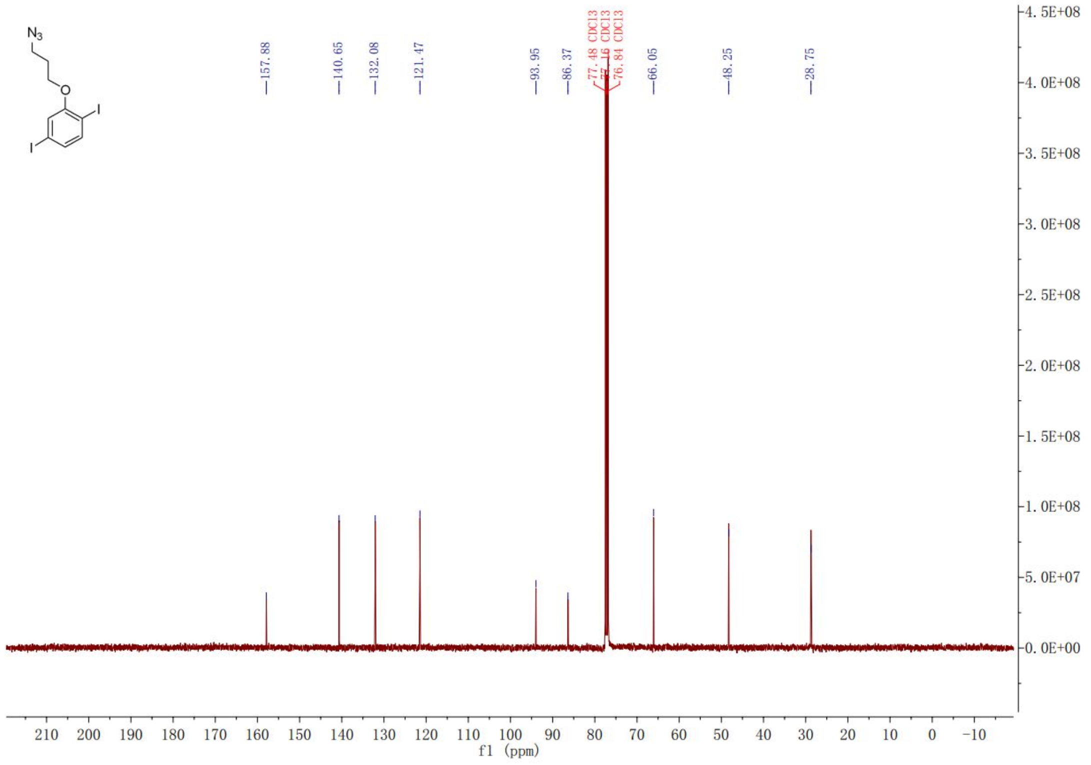

Figure S27. ^{13}C NMR of $\mathbf{S_1}$.

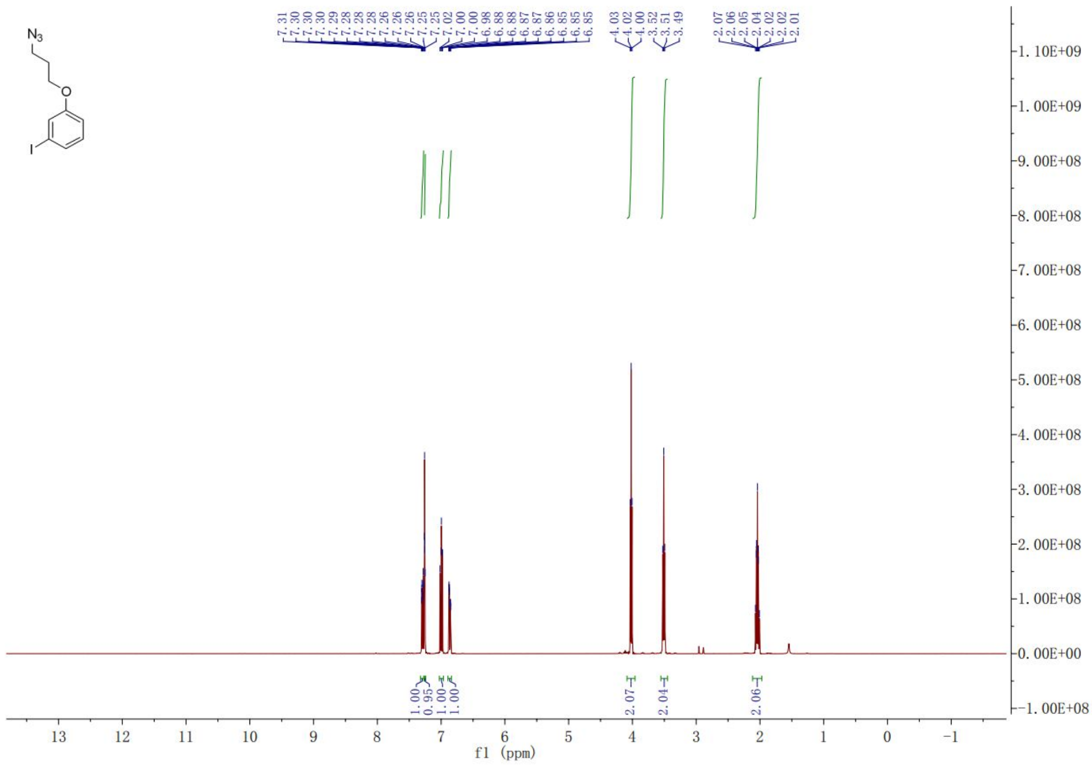

Figure S28. ^{1}H NMR of $\mathbf{S_2}$.

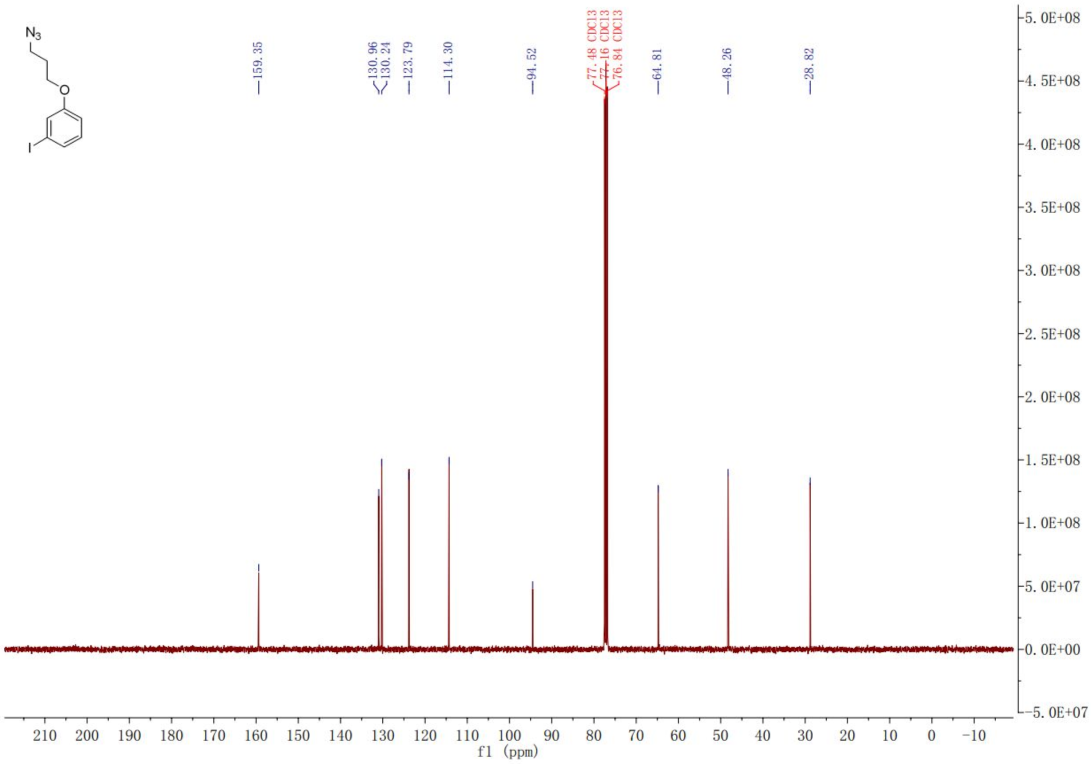

Figure S29. ^{13}C NMR of $\mathbf{S_2}$.

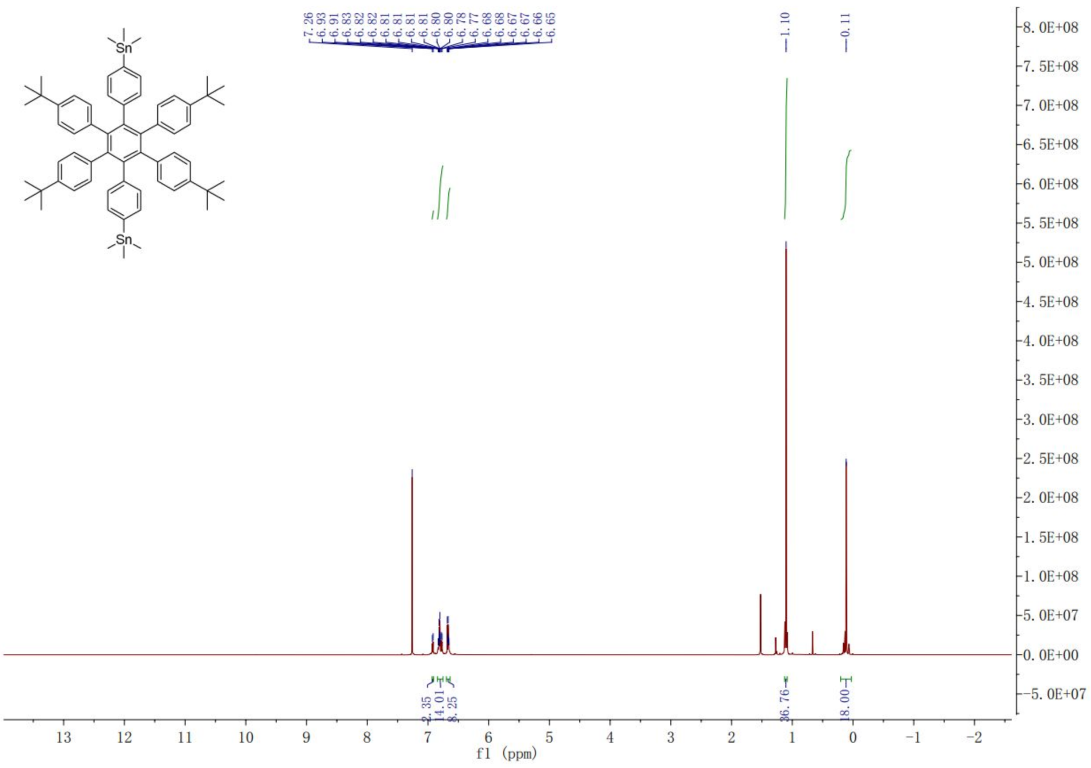

Figure S30. ^{1}H NMR of $\mathbf{T_1}$.

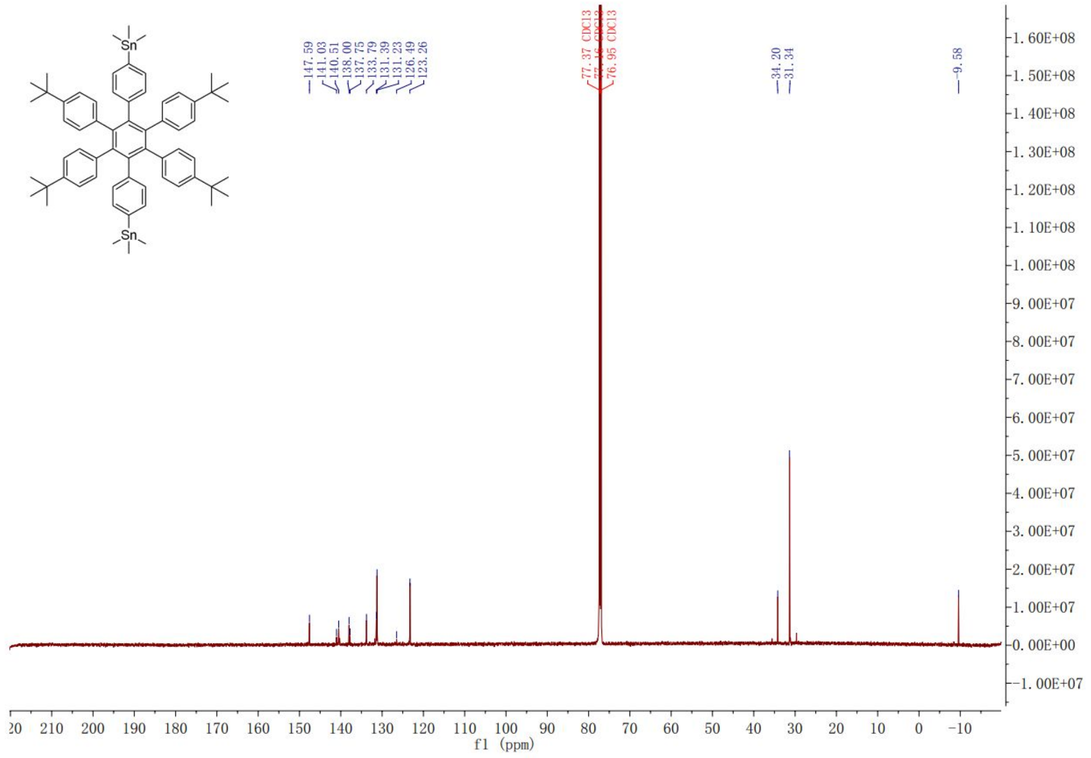

Figure S31. ^{13}C NMR of $\mathbf{T_1}$.

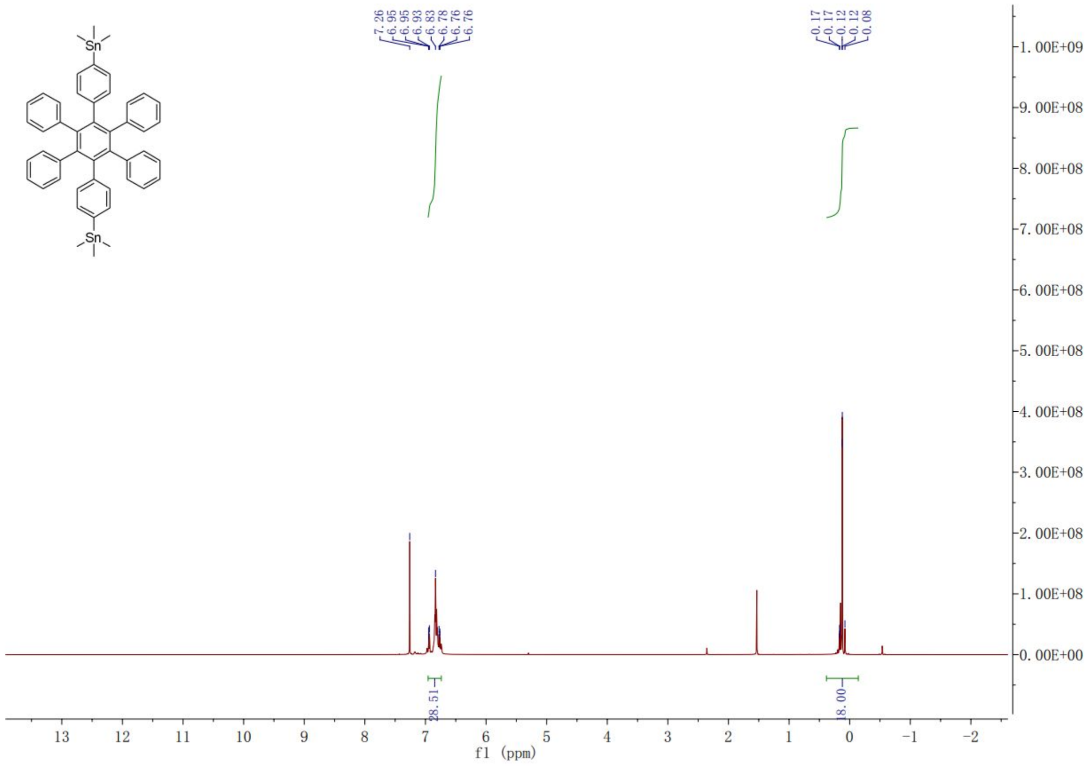

Figure S32. ^{1}H NMR of $\mathbf{T_2}$.

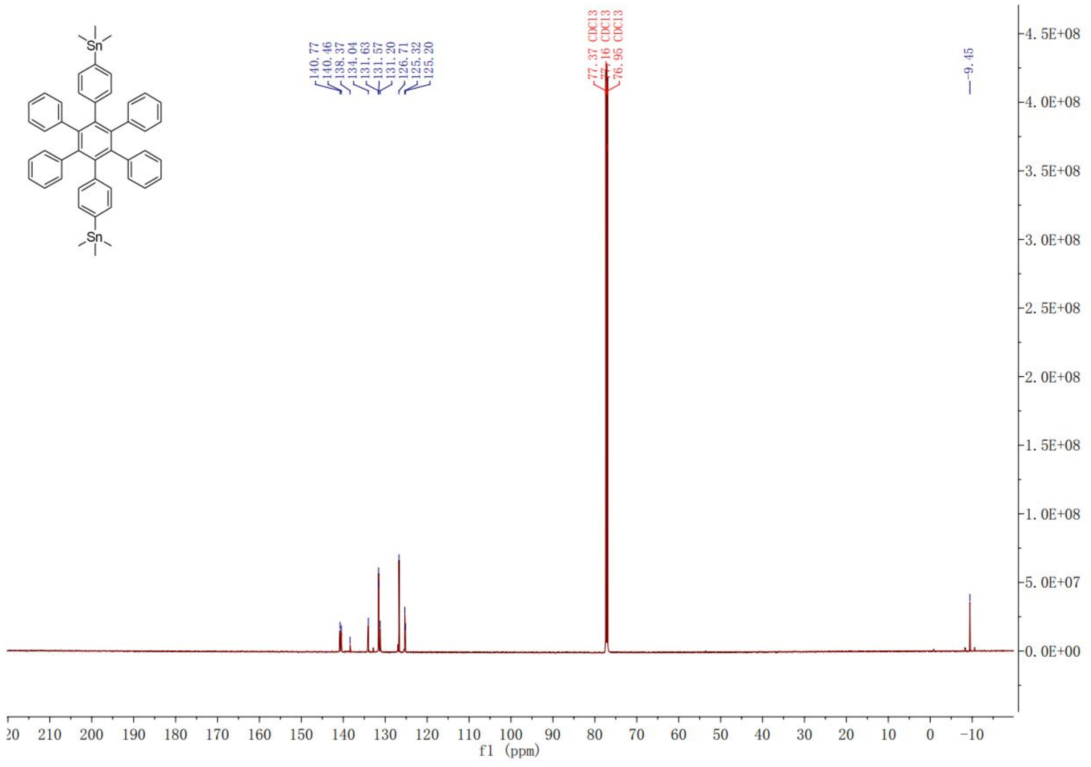

Figure S33. ^{13}C NMR of $\mathbf{T_2}$.

References

1. Kim, C.; Wallace, J. U.; Chen, S. H.; Merkel, P. B., Effects of Dilution, Polarization Ratio, and Energy Transfer on Photoalignment of Liquid Crystals Using Coumarin-Containing Polymer Films. *Macromolecules* **2008,** *41* (9), 3075-3080.

2. Diez-Perez, I.; Li, Z.; Hihath, J.; Li, J.; Zhang, C.; Yang, X.; Zang, L.; Dai, Y.; Feng, X.; Muellen, K.; Tao, N., Gate-controlled electron transport in coronenes as a bottom-up approach towards graphene transistors. *Nature Communications* **2010,** *1* (1), 1-5.

3. Yang, X.; Dou, X.; Müllen, K., Efficient Synthesis of Symmetrically and Unsymmetrically Substituted Hexaphenylbenzene Analogues by Suzuki–Miyaura Coupling Reactions. *Chemistry – An Asian Journal* **2008,** *3* (4), 759-766.
